# Conceptual and methodological advances in habitat‐selection modeling: guidelines for ecology and evolution

**DOI:** 10.1002/eap.2470

**Published:** 2021-11-28

**Authors:** Joseph M. Northrup, Eric Vander Wal, Maegwin Bonar, John Fieberg, Michel P. Laforge, Martin Leclerc, Christina M. Prokopenko, Brian D. Gerber

**Affiliations:** ^1^ Wildlife Research and Monitoring Section Ontario Ministry of Northern Development, Mines, Natural Resources and Forestry Peterborough Ontario K9L 1Z8 Canada; ^2^ Environmental and Life Sciences Graduate Program Trent University Peterborough Ontario K9L 1Z8 Canada; ^3^ Department of Biology Memorial University of Newfoundland St. John's Newfoundland A1B 3X9 Canada; ^4^ Department of Fisheries, Wildlife and Conservation Biology University of Minnesota St. Paul Minnesota USA; ^5^ Département de Biologie Caribou Ungava and Centre d'études nordiques Université Laval Québec Québec G1V 0A6 Canada; ^6^ Department of Natural Resources Science University of Rhode Island Kingston Rhode Island USA

**Keywords:** animal movement, habitat selection, integrated step‐selection analysis, movement ecology, point process, radio collar, resource‐selection function, step‐selection function, telemetry, use‐available design, wildlife

## Abstract

Habitat selection is a fundamental animal behavior that shapes a wide range of ecological processes, including animal movement, nutrient transfer, trophic dynamics and population distribution. Although habitat selection has been a focus of ecological studies for decades, technological, conceptual and methodological advances over the last 20 yr have led to a surge in studies addressing this process. Despite the substantial literature focused on quantifying the habitat‐selection patterns of animals, there is a marked lack of guidance on best analytical practices. The conceptual foundations of the most commonly applied modeling frameworks can be confusing even to those well versed in their application. Furthermore, there has yet to be a synthesis of the advances made over the last 20 yr. Therefore, there is a need for both synthesis of the current state of knowledge on habitat selection, and guidance for those seeking to study this process. Here, we provide an approachable overview and synthesis of the literature on habitat‐selection analyses (HSAs) conducted using selection functions, which are by far the most applied modeling framework for understanding the habitat‐selection process. This review is purposefully non‐technical and focused on understanding without heavy mathematical and statistical notation, which can confuse many practitioners. We offer an overview and history of HSAs, describing the tortuous conceptual path to our current understanding. Through this overview, we also aim to address the areas of greatest confusion in the literature. We synthesize the literature outlining the most exciting conceptual advances in the field of habitat‐selection modeling, discussing the substantial ecological and evolutionary inference that can be made using contemporary techniques. We aim for this paper to provide clarity for those navigating the complex literature on HSAs while acting as a reference and best practices guide for practitioners.

## Introduction

Animal habitat selection is a behavior that has commanded the focus of ecological and evolutionary research for a half century (Fretwell and Lucas 1969, Shafer et al. 2014). The study of habitat selection is critical for understanding how individual animals interact with their environment to produce population‐level patterns of distribution and abundance (Boyce and McDonald 1999, Matthiopoulos et al. 2015). Habitat selection also drives an array of important ecological and evolutionary processes including trophic structuring (Ford et al. 2014), spatial patterns of relatedness and dispersal (Shafer et al. 2012) and the formation of ecological traps (Robertson et al. 2013). Furthermore, understanding the relationship between species and their habitat is fundamental for a range of problems in applied ecology, including assessing and predicting effects of climate and land‐use change (Sohl 2014), modeling disease dynamics (Tardy et al. 2014) and informing the design of protected areas (Guisan et al. 2013). Therefore, understanding the process of habitat selection and clarifying the methods for quantifying this process are of critical importance to the study of animal ecology and evolution and to the conservation and management of species.

**Fig. 1:**
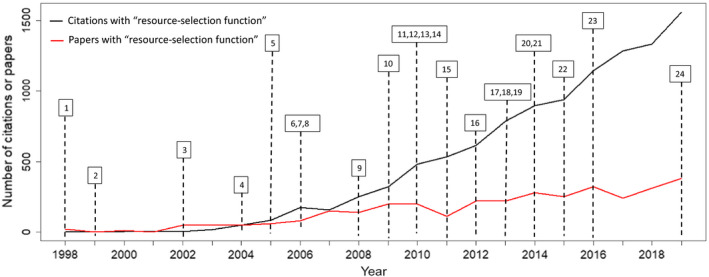
Number of citations and publications annually for papers with keywords “resource‐selection function,” the type of selection function most commonly applied in the literature, over time based on a Web of Science search. Numbers reference citations below, which comprise some of the key advancements made in modeling resource selection, helpful reviews, or highly cited papers. ^1^Mysterud and Ims (1998*a*), ^2^Boyce and McDonald (1999), ^3^Boyce et al. (2002), ^4^Keating and Cherry (2004), ^5^Fortin et al. (2005), ^6^Gillies et al. (2006), ^7^Johnson et al. (2006), ^8^Lele and Keim (2006), ^9^Johnson et al. (2008), ^10^Forester et al. (2009), ^11^Fieberg et al. (2010), ^12^McLoughlin et al. (2010), ^13^Warton and Shepherd (2010), ^14^Beyer et al. (2010), ^15^Matthiopoulos et al. (2011), ^16^Aarts et al. (2012), ^17^Northrup et al. (2013), ^18^Johnson et al. (2013), ^19^Lele et al. (2013), ^20^Hooten et al. (2014), ^21^van Beest et al. (2014*b*), ^22^Matthiopoulos et al. (2015), ^23^Avgar et al. (2016), ^24^Muff et al. (2020).

Recent technological advancements in animal tracking (Kays et al. 2015), coupled with diverse and flexible statistical techniques (Elith and Leathwick 2009*a*, Hooten et al. 2017), and ever‐increasing computational power, offer the opportunity for unprecedented insight and inference to the habitat‐selection patterns of animals. These advances have led to a considerable increase in the number of studies developing and refining habitat‐selection theory and statistical models for quantifying habitat selection. Chief among the methods used to make inference to habitat‐selection patterns is a suite of models referred to as selection functions (e.g., resource‐selection functions, step‐selection functions; Manly et al. 1993, Boyce and McDonald 1999, Fortin et al. 2005). References to these models in the peer‐reviewed literature have increased dramatically since the turn of the century (Fig. 1), and although their application has led to ecological insights for individual studies, this work has largely been conducted in the absence of a coherent framework that connects ecological concepts or theory with advanced statistical modeling and inference (but see Matthiopoulos et al. 2020). As a result, the literature on this topic is disjointed, making it difficult to draw general conclusions about ecological (e.g., McLoughlin et al. 2010) and evolutionary processes (Fortin et al. 2008, Shafer et al. 2012, Leclerc et al. 2016) related to habitat selection.

Currently, guidance on how to address the most important methodological and conceptual issues when conducting habitat‐selection analyses (HSAs) exists in piecemeal throughout the literature. Furthermore, new statistical methods are often developed without considering ecological theory or practical applications. The result is two‐fold: (1) the methods and concepts important to studying habitat selection are becoming inaccessible or even irrelevant to researchers aiming to use these models to address questions in ecology and evolution, and (2) the lack of a coherent understanding of the assumptions and implications of modeling decisions can lead to spurious results, which threaten basic science and may negatively impact management and conservation efforts. Therefore, there is a clear need to take stock of our current state of knowledge underlying the approaches most often used to assess habitat selection, and to provide guidance on applying these methods when making inference to ecological and evolutionary processes. Such guidance can provide more unity among approaches and will facilitate better science, but also could improve comparisons among studies.

**Fig. 2:**
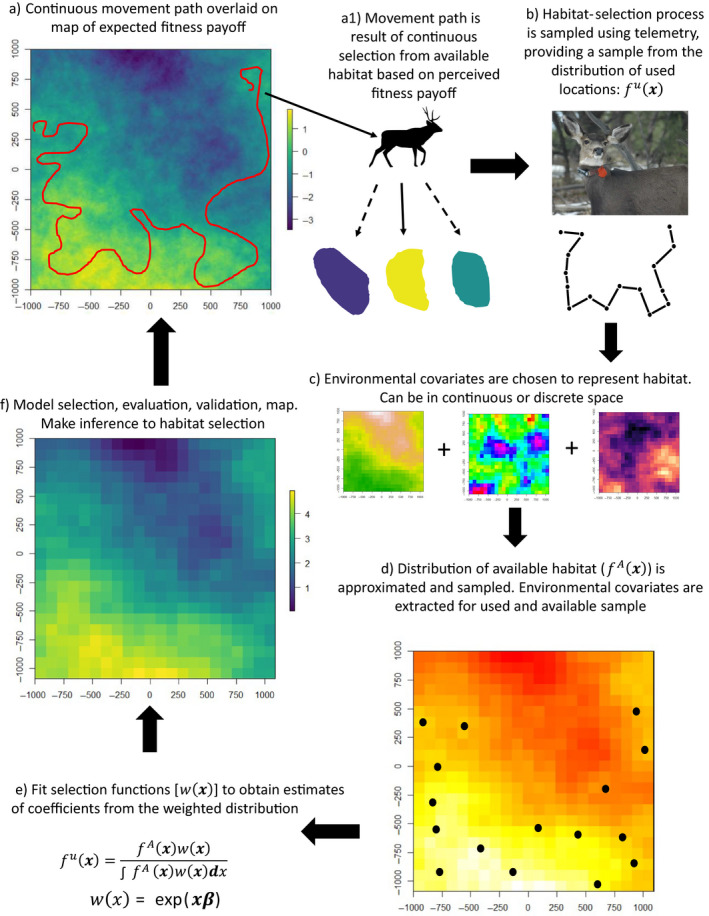
Overview of the ecological and analytical process underlying habitat‐selection analyses (HSAs). HSAs are motivated by a variety of questions. Although the general framework, detailed graphically above and expanded on below, and most of the analytical steps are similar, the question of interest dictates many of the specific details. For each panel, above, we direct the reader to the relevant section of the paper in which the topic is discussed. (a) The true process underlying the habitat‐selection patterns of animals. We refer to the map underlying habitat‐selection decisions as the expected fitness payoff. This map will be correlated with and in some cases equivalent to an individual's utilization distribution or the steady‐state density of animals (Moorcroft and Barnett 2008, Signer et al. 2017). *What is an HSA?* and *How Do Selection Functions Provide Inference to Habitat Selection?* discuss the processes underlying HSAs. (b) The process of habitat selection is typically inferred from discrete locations sampled along the animal's movement path using telemetry. The resulting data are a sample of the continuous distribution of used locations (see *The use distribution*). The sampling design can induce multiple types of statistical dependence including within‐individual serial autocorrelation in location data, or from the repeat sampling of individual animals within a population (see *Statistical dependence in studies of habitat selection*). The fix rates and duration of sampling are largely under the control of the analyst, with factors such as sex and age composition also partially under analyst control. Decisions about these factors are fundamental to questions about sex and age dependence (*Context dependence in habitat selection*), scale (*Scale dependence*), environmental context dependence (i.e., functional responses; *Functional responses*), density dependence (*Density dependence*), and individual variation (*Individual variability*). Furthermore, because the data used in HSAs are sampled from the movement path, movement should be considered (*Movement and HSAs*). Habitat‐selection behavior is likely to be highly context dependent and therefore the many sources of dependence in this behavior need to be accounted for, either technically or inferentially (*Context dependence in habitat selection*). Many decisions made at this stage of the analysis have the potential to introduce uncertainty (*Uncertainty, mapping and inference*). (c) Environmental variables, typically represented spatially, are chosen to represent habitat for the species of interest. Although the data themselves are points, and typically analyzed in environmental space (see Glossary of terms Table 1), technically, environmental covariates are often represented as pixels in a GIS (see *Habitat*). There is typically inherent spatial autocorrelation in environmental covariates, but this may not be of major concern for HSAs (see *Statistical dependence in studies of habitat selection*). The analyst must decide on whether to represent environmental covariates in discrete (i.e., as pixels) or continuous space (or a mix) and the extent, grain and resolution (only for discrete space) of the covariates (see *Scale dependence*). Representation of environmental covariates using remotely sensed data can introduce uncertainty into the analysis (*Uncertainty, mapping and inference*). (d) Unique combinations of environmental variables represent habitat types (see *Habitat*). These environmental variables can be broken down into resources, risks and conditions (see Glossary of terms Table 1), can be static or dynamic and can account for a range of dependencies in behavior (see *Context dependence in habitat selection*). Covariates are extracted at used locations. Locations available for the animal to select are sampled using a suite of potential methods, across multiple scales of interest, and environmental covariates are summarized (*The available distribution*, *Concepts and Advances: The available distribution* and *Scale dependence*). The available distribution is under control of the analyst and can be chosen to address questions on scale of habitat selection (*Scale dependence*), environmental context dependence (*Functional responses*) and density dependence (*Density dependence*). Many options exist for incorporating movement directly into the definition of availability (*Movement and HSAs*). The choice of how to quantify availability can introduce uncertainty into the analysis (*Uncertainty, mapping and inference*). (e) Selection functions (see Glossary of terms Table 1) are fit using a range of statistical algorithms that all ultimately provide an estimate of coefficients from a weighted distribution (*Selection functions*, *How are selection functions fit?* and *How Do Selection Functions Provide Inference to Habitat Selection?*). If there are any dependencies in the data that are of interest, such as density dependence, they should be accounted for at this step (*Context dependence in habitat selection* and *Overview of Habitat‐Selection Analyses*). The various sources of uncertainty should be accounted for in this stage if possible (*Uncertainty, mapping and inference*). (f) Depending on the questions of interest, model selection is performed, models are evaluated and validated, and maps are produced to visualize results (*Uncertainty, mapping and inference* and *Model selection, evaluation and validation*). If any covariates used in the analysis are calculated in continuous space, they must be discretized for mapping. Inference is then made to habitat selection (*Selection functions* and *How Do Selection Functions Provide Inference to Habitat Selection?*), while considering the variety of sources of uncertainty (*Uncertainty, mapping and inference*). All inference is conditional on the available distribution and all other dependencies in the data (*The available distribution*, *Concepts and Advances: The available distribution* and *Context dependence in habitat selection*). Dependencies that fundamentally influence the inference from HSAs include scale dependence (*Scale dependence*), environmental context dependence (i.e., functional responses; see *Functional responses*), density dependence (*Density dependence*) and dependence occurring from individual variation (*Individual variability*). Although many of these factors can, and often should, be incorporated at the analysis stage, at a minimum they must be considered when making inference. Any sources of uncertainty that are not formally accounted for in the analysis stage must be addressed when making inference and visualizing results (see *Uncertainty, mapping and inference*). The result of an HSA can be visualized in different ways (see *Uncertainty, mapping and inference*).

We aimed to provide an overview of the current state of the field of modeling habitat selection by: (1) providing an accessible review of the history and development of the frameworks most commonly used to conduct HSAs, (2) synthesizing the most significant and/or recent methods and conceptual advances, and (3) discussing important assumptions and their implications for inference. Here, we used HSA as a broad term to capture the suite of commonly used approaches for understanding habitat selection, including resource‐selection functions (RSFs), step‐selection functions (SSFs) and integrated step‐selection analyses (iSSAs). We focused on HSAs conducted under a use–availability design applied to animal location data obtained from telemetry technologies. These analyses comprise the vast majority of recent published works examining habitat selection and have been the focus of most recent methodological advances. We note that use–availability approaches (see *Overview of Habitat‐Selection Analyses*) can be applied to other types of data (e.g., snow tracking or aerial survey data; see Manly et al. 1993). The broad analytical framework under which most of these HSAs are conducted is identical to that used in analysis of presence‐only data to fit species distribution models, such as museum records (i.e., a Poisson point process typically is assumed to underlay both types of data; Warton and Shepherd 2010, Aarts et al. 2012, Fithian and Hastie 2013, Johnson et al. 2013, McDonald et al. 2013, Northrup et al. 2013). Therefore, the general concepts we discuss have broader applicability than just to models fit to telemetry data. However, many of the specific analytical approaches are unique to telemetry data and therefore warrant targeted consideration. We begin with a brief background on HSAs, clarifying concepts that, in our experience, are not well understood. We then discuss the inferences that can be drawn from HSAs. In the sections that follow, we do not aim to provide an exhaustive review of the available literature but to discuss important aspects of HSAs as well as recent and ongoing advances. To guide readers through this text, we offer a figure describing the conceptual and technical aspects of HSAs that can be used to reference specific sections of this review (Fig. 2).

**Table 1:** Glossary of terms.

Term	Definition
Available distribution	Probability density function characterizing the relative frequency of locations that are accessible to the animal. Can be defined in geographic or environmental space
Available locations	A sample of locations from the available distribution
Condition	Environmental variable influencing the functioning of the organism of interest, e.g., temperature or humidity
Environmental space	The space whose dimensions are environmental variables
Environmental variable	A measurable characteristic of the environment that, for the species of interest, could represent a condition, resource or risk. Note that, technically, we rarely measure these precisely in HSAs and they are often represented by remotely sensed data products
Extent	The complete areal or temporal coverage of an analysis
Geographic space	The space defined by physical dimensions (e.g., latitude, longitude)
Grain	The area or time period surrounding a point or observation over which ecological variables are considered
Habitat	A point in environmental space, defined by a set of conditions, resources and risks for the species of interest. Although habitat is approximated by the set of environmental covariates measured in an analysis, the full suite of conditions resources and risks for a species are rarely known or measured. Furthermore, the specific relationship between habitat and the fitness of an animal is density dependent
Habitat selection	The process through which individual animals differentially use habitats relative to their availability at a given population density; habitat selection, by definition, is density dependent
Habitat type	Any unique combination of environmental variables (conditions, resources and risks) representing habitat for the species of interest. For HSAs applied to telemetry data, this is typically the unique combination of environmental covariates at a point or within a pixel
Habitat unit	Discrete, analyst‐defined areas, in geographic space, over which the environmental variables representing habitat are quantified
Habitat use	The proportion of time an animal spends in a habitat unit
Occurrence or occupancy	The physical presence of an animal at a location
Preference	Habitat selection conditional on all habitat types being equally available
Resolution	The minimum mapping or temporal unit of data
Resource	A substance, object or place required by the focal organism for growth, maintenance and reproduction at a given population density. The quantity of a resource may be reduced by the organism. Resources strictly relate positively to fitness of the organism
Risks	Environmental variables that are negatively related to fitness at a given population density. That is, they reduce the probability of survival or reproduction
Selection function	A weighting function describing the relative probability of selecting a location or unit, based on environmental covariates
Use distribution	Probability density function characterizing the relative frequency of locations that are used by the animal. Can be defined in geographic or environmental space. When defined in geographic space, it is often referred to as a utilization distribution (UD)
Used locations	A sample of locations from the use distribution

Notes

We generally borrow many definitions from Matthiopoulos et al. (2020). Interested readers should see their glossary for more detail.

## Overview of Habitat‐selection Analyses

Several publications provide helpful overviews of HSAs, selection functions, and the related terminology (e.g., Manly et al. 1993, Boyce and McDonald 1999, Johnson et al. 2006, Beyer et al. 2010, Johnson et al. 2013, Lele et al. 2013, Matthiopoulos et al. 2020, Fieberg et al. 2021). These works have proven critical for creating the foundation upon which HSAs have become so popular; however, some of these works are beginning to become outdated, considering recent conceptual and methodological advances, while others are difficult for practitioners to digest, due to their statistical focus. Furthermore, we have noted a recent conceptual divide in the literature developing the theory and statistical methods of HSAs, with some researchers adopting the terminology of point process models (e.g., Aarts et al. 2012, Hooten et al. 2017, Muff et al. 2020, Fieberg et al. 2021), others maintaining the more traditional terminology outlined by Manly et al. (1993; e.g., Lele et al. 2013), and still others attempting to provide a more general set of terms that span analyses conducted using telemetry data and more traditional species distribution models (Matthiopoulos et al. 2020). The resulting literature is potentially confusing for practitioners or those new to HSAs. Therefore, we provide a brief overview and definitions for HSAs and selection functions. We refer readers to the Glossary (Table 1) and conceptual figure (Fig. 2) when navigating the paper. In our opinion, much of the confusion in the literature on HSAs arises from two sources: (1) the variety of terminology used to describe HSA components, and (2) the history of debate about appropriate methods to fit them; we address these topics in turn. Throughout the following material, we outline a variety of assumptions and analytical decisions that are required when conducting the most common types of HSAs. There are many interconnections among topics, and therefore, we note that we will regularly refer readers between subsections for subsequent details (see Fig. 2 also for navigation purposes). Furthermore, we note that many terms we use and concepts we define are used differently in the literature. In the sections that follow, we attempt to link our definitions and concepts to other uses in the literature to bridge the terminology divide that exists in this field.

### What is an HSA?

Conceptually, we define an HSA as any analysis that attempts to capture the following, highly simplified process: as an animal encounters available habitat, they select habitat and use it in some way. Any HSA consists of four components that we discuss in the sections that follow: (1) a precise, technical definition of habitat, (2) a probability density function quantifying the distribution of available habitat, (3) a probability density function quantifying the distribution of used habitat, or, more commonly, a sample from that distribution, and (4) a function, referred to as a selection function, quantifying selection of habitat relative to availability.

### Habitat

The term habitat has been variably defined in the literature, both conceptually and technically (Morris 2003). Many foundational works define habitat in terms of discrete spatial units where the species of interest is known to or could occur (Morris 2003). In contrast, Matthiopoulos et al. (2020) defined habitat as a point in environmental space, which they argue is more cohesive with the way in which HSAs are functionally performed. They point out that defining habitat in discrete space, as opposed to as a point, raises some issues analytically. First, the probability that a discrete unit will be used increases with the size of the unit and with the sampling duration. Therefore, the results of HSAs are highly sensitive to the definition of discrete space. Furthermore, species perceive many components of the environment in a continuous way, and, in HSAs, we may want to define specific environmental variables continuously (e.g., distance to some feature), which is only possible for points. Defining habitat in continuous space offers more analytical flexibility. Largely following Matthiopoulos et al. (2020), here, we define habitat as a point in environmental space, defined by a set of conditions, resources and risks for the species of interest (see Glossary of terms in Table 1). The notable difference between this definition and that of Matthiopoulos et al. (2020) is that they do not focus their definition on a species of interest. Although ignoring species provides maximal flexibility in its conception, it breaks with foundational definitions of habitat. We believe our definition is a suitable balance, maintaining the technical flexibility of Matthiopoulos et al. (2020) while keeping consistent with the foundational literature on habitat and habitat selection. This definition of habitat is similar to how Lele et al. (2013) and others define “resources.” Furthermore, we note that it is common to refer to “resource‐selection functions” when conducting HSAs. However, we prefer the term habitat because it better captures the goal of HSAs, which is to infer to the process of habitat selection; introducing an intermediate term, such as resources, can lend confusion, in our opinion. We note that, conceptually, habitat for a species represents all the environmental variables that influence the fitness of the animal at a given point (i.e., all conditions, resources and risks). However, technically, we are never able to quantify all influences, and therefore the analyst‐defined habitat is only an approximation of the way in which the species experiences its environment.

Technically, in an HSA, habitat is defined by user‐chosen environmental covariates measured at a point, or, commonly within analyst‐defined pixels (making the HSA an analysis in discrete space) in a geographic information system (GIS). Most often, these pixels match the resolution of available remotely sensed data. Variables can include any measurable quantity in environmental space, such as elevation, vegetation class (e.g., shrub dominated), or even dynamic processes such as predator or prey distributions, conspecific density, or social environment. In statistical terms, whether treated discretely or continuously, environmental variables representing habitat are depicted as a matrix of covariates with the number of columns equal to the number of covariates and the number of rows equal to the number of used and available points (see *The available distribution*); represented mathematically as x. The attributes of habitat can be positively or negatively associated with the use of the unit by the animal, and therefore its occurrence at a point or within a pixel (discussed briefly by Beyer et al. 2010).

### The available distribution

The available distribution is a probability density function characterizing the relative frequency of locations (in geographic space) or environmental variables (in environmental space) that are accessible to the individuals under study. The available distribution is often assumed (implicitly) to be the sampling frame over geographic or environmental space, and possibly time, from which animals have selected habitat (in geographic space, a uniform probability is typically assumed over the sampling frame, Hooten et al. 2017). It is often written in statistical notation as $f^{A}\left(x\right)$, when working in environmental space, or $f^{A}\left(x\left(s\right)\right)$, when working in geographic space, with x referring to a matrix of environmental covariates and *f* is a probability density function (e.g., uniform throughout a home range). Defining what is available to the animal is a fundamental model assumption of an HSA (Matthiopoulos 2003, Beyer et al. 2010, Matthiopoulos et al. 2011, Aarts et al. 2013, Northrup et al. 2013, Paton and Matthiopoulos 2016). Its definition is up to the analyst and determines the type of inference gained. It is commonly defined in a number of ways, ranging from a simple bounding box around a study area or home range (Ciarniello et al. 2007) to complex, movement‐based probability distributions (e.g., Hooten et al. 2014, Northrup et al. 2015, Avgar et al. 2016). Implicit in any definition of the available distribution is the assumption that all habitat in this distribution is physically accessible to the animal (Paton and Matthiopoulos 2016), although sometimes model covariates may be used to further modify accessibility (e.g., to exclude areas that require crossing a known barrier). We direct readers to the more detailed discussion of the available distribution in *Concepts and Advances: The available distribution*.

### The use distribution

The use distribution is a probability density function characterizing the relative frequency of locations (in geographic space) or environmental variables (in environmental space) that are used by the animal. The use distribution is often written as $f^{u}\left(x\right)$, in environmental space, or $f^{u}\left(x\left(s\right)\right)$, in geographic space, indicating it is a function of the environmental covariates, x, describing habitat at spatial location s. Because we only know the location of an animal fit with a GPS collar at scheduled, or sometimes irregular, times (e.g., every hour, every day), we only observe a sample of this distribution, i.e., the telemetered locations. However, the distribution itself is continuous (Hooten et al. 2014) and in fact, when defined in geographic space, is equivalent to the utilization distribution over the sampling period (Matthiopoulos et al. 2020 Chapter 3, Fieberg et al. 2021). These samples represent the collection of habitats that coincides with where an animal occurred at the time of successful GPS fixes. We make the simplifying assumption that if an animal is present at a habitat location then it is using it in some way. Note that the nature of this use can vary, for example, depending on if the animal is foraging vs. resting or seeking refuge from predators, therefore potentially complicating the interpretation of the inference gained from HSAs.

### Selection functions

The term “selection function” predates its use in habitat‐selection studies and is defined as a function that describes how one probability distribution is translated into another probability distribution (Manly 1985, McDonald and Manly 1989, McDonald et al. 1990*b*, McDonald et al. 1995). Manly (1985) and later McDonald et al. (1995) describe the use of selection functions in modeling natural selection, whereby a population with some distribution of phenotypes changes to a population with a different distribution of phenotypes based on the selection (i.e., fitness) function. Manly et al. (1993) show how this concept can be applied in HSAs and popularized the term “resource‐selection function”. Researchers have continued to develop a range of approaches that use the terminology of a selection function, including SSFs (Fortin et al. 2005) and energy selection functions (Klappstein et al. 2020). Here, we use a slightly narrower definition of selection function that is specific to HSAs: a weighting function describing the relative probability of selecting a location or unit, based on environmental covariates. However, the general idea developed in the foundational literature (Manly 1985, McDonald and Manly 1989, McDonald et al. 1990*b*, *1995*) holds; the selection function translates a probability distribution describing habitat that is available to an animal into a probability distribution describing the habitat used by the animal. The selection function itself is commonly written as w(x), again indicating it is a function of the environmental covariates describing habitat (x), but precisely what selection functions are is still confusing to many. Manly et al. (1993) defined the RSF as any function proportional to the probability of use of a resource unit. In this case, resource units essentially match our definition of habitat, but in discrete space. Lele and Keim (2006) discussed that Manly's definition of a selection function is problematic because the probability that a discrete spatial unit will be used depends on the size of the unit and the duration of the study. Furthermore, a probability cannot be associated with a point, which is dimensionless, and therefore this definition is not amenable for modeling in continuous space. Lele et al. (2013) clarified that selection functions are actually measuring the behavioral process of selection. Therefore, they refer specifically to RSFs as any function proportional to the probability of selection of a resource unit (again similar to our definition of habitat in discrete space). Furthermore, clarifying the statistics underlying selection functions, Warton and Shepherd (2010) showed that the modeling approaches used in traditional HSAs (i.e., a selection function when availability is static) are equivalent to an inhomogeneous Poisson point process (IPP), which is a model for random points in space where the expected density of these points is described by spatial covariates (see also Aarts et al. 2013, Fithian and Hastie 2013, Fieberg et al. 2021). This equivalency suggests that the traditional selection function is itself equivalent to the intensity of the IPP. However, this intensity function quantifies the expected density of points, while in telemetry studies, this quantity is at least partially under control of the analyst; that is, the density of points increases with a more frequent fix schedule, or with a greater number of collared individuals. Therefore, several papers have pointed out that in most HSAs, the intercept in the selection function represents the ratio of used to available points when all other covariates are set to 0 and therefore is biologically meaningless (Johnson et al. 2006, Muff et al. 2020, Fieberg et al. 2021). Dropping the intercept, the selection function represents the relative (i.e., not absolute) intensity of the IPP or the relative selection strength (Aarts et al. 2013, Avgar et al. 2017, Hooten et al. 2017, Fieberg et al. 2021). For those new to HSAs, this variable terminology can be confusing. However, it is sufficient simply to understand that HSAs provide inference to the selection behavior of animals. This point is critical, as it identifies HSAs as an approximation of the behavioral patterns of an animal. Therefore, HSAs can provide inference to the mechanisms generating patterns of habitat use, although one must carefully consider scale dependence (*Scale dependence*), environmental context dependence (*Functional responses*), and density dependence (*Density dependence*) when interpreting parameters in HSAs. Furthermore, the weighted distribution theory upon which selection functions are based (see *How are selection functions fit?* for further details) allows the prediction of the expected density of use over a sampled time period within defined spatial units, or the utilization distribution (Signer et al. 2017, Fieberg et al. 2021).

We think that the above methodological and conceptual discussion can be overwhelming and confusing to those new to this field. Perhaps even more confusing is the discussion of the utility of methods for estimating the absolute probability of selection of habitat units (i.e., resource‐selection probability functions or RSPFs; Lele and Keim 2006, Lele 2009) vs. selection functions that are, by definition, a relative measure of selection. RSPFs make most sense when modeling discrete sample units that are observed to be either used or not used. Although Lele and Keim (2006) and Lele (2009) developed methods for estimating RSPFs with use–availability data, Hastie and Fithian (2013) later showed that these approaches require untenable assumptions about the selection function, namely that it is *exactly* linear on the link scale, and that estimates of absolute probabilities are not robust to violations of this assumption. Therefore, we do not discuss these methods further.

### How are selection functions fit?

There has been much debate in the literature over the proper methods used to fit selection functions in a use–availability framework. We think that the primary source of debate, and confusion for researchers new to HSAs, is the analysis of use–availability data using logistic regression. Although there are a multitude of frameworks available for modeling selection functions (see Johnson et al. 2008, Lele 2009, Matthiopoulos et al. 2011, Fithian and Hastie 2013, Hooten et al. 2014, Muff et al. 2020), logistic regression is by far the most commonly applied. Models fit in this manner assign locations where an animal occurred (e.g., GPS fixes; the used sample) as 1s and require that the analyst sample the available distribution, often by generating random locations that are then assigned 0s. There are several methods for sampling the available distribution, including systematic sampling or even a census of all pixels in a GIS. At face value, the creation of this “available” or “pseudo‐absence” data seems worrisome, as the analyst has complete discretion over the creation of data and it very likely contains instances of used habitat being classified as 0s. This problem of “contamination” and other concerns were brought up by Keating and Cherry (2004), in what now has become a foundational critique of HSAs. However, these criticisms have been shown to be largely irrelevant, as the use of logistic regression is solely for computational convenience, as discussed in several recent works (Johnson et al. 2006, Warton and Shepherd 2010, Aarts et al. 2012, Fithian and Hastie 2013, Northrup et al. 2013, Hooten et al. 2017, Fieberg et al. 2021). We briefly review the main points. Early HSAs were based on weighted distribution theory (McDonald et al. 1990*a*, Patil 2002), whereby the distribution of used habitats ($f^{u}\left(x\right))$ is a function of the distribution of available habitat ($f^{A}\left(x\right))$, weighted by the selection function ($w\left(x\right)$) (see Lele and Keim 2006 for a more complete discussion). Mathematically, this is written as:

$$f^{u}\left(x\right)=\frac{f^{A}\left(x\right)w\left(x\right)}{\int f^{A}\left(x\right)w\left(x\right)dx}$$

In this equation, the denominator is an integral over the domain, in environmental space, of all used and available habitat (**x**), providing a constant that ensures a proper probability distribution (i.e., all values of $f^{u}\left(x\right)$ must be positive and the area under this curve must equal one). Warton and Shepherd (2010) and later, in more detail, Aarts et al. (2012), showed that if we assume the selection function takes the exponential form—i.e., $w\left(x\right)=\exp(x\beta$), where β represents a vector of regression coefficients indicating selection or avoidance of a particular environmental variable—the likelihood for the weighted distribution is identical to an IPP in continuous space. It is also possible to approximate the point process in discrete space, leading to a Poisson regression model (Aarts et al. 2012, Matthiopoulos et al. 2020). Furthermore, coefficients obtained from logistic regression asymptotically approximate the coefficients in the selection function of the weighted distribution. That is, as the number of randomly generated available locations approaches infinity, the coefficients from logistic regression approach the coefficients from the weighted distribution. These issues were initially presented by Johnson et al. (2006), but the asymptotic equivalence between the coefficients obtained from logistic regression and a Poisson point process has further clarified this discussion.

This connection between logistic regression and a Poisson point process has several practical implications. First, randomly generating availability samples and using these in a logistic regression is a computational trick to approximate the Poisson point process and is a completely legitimate procedure. Of note, when using logistic regression, one must still assume that the selection function takes the exponential form, i.e., $w\left(x\right)=\exp(x\beta$), so even though coefficients can be estimated using a logit link $\left(\frac{\exp\left(x\beta \right)}{1+\exp\left(x\beta \right)}\right)$, which is the standard link function for binary regression models in most software programs, the exponential form must be used when mapping selection or making predictions (i.e., exponentiating the linear combination of covariates and coefficients while excluding the intercept). This also means that users must take caution when using functions in statistical software for making predictions, such as the *predict* function in R, to ensure the proper transformation is used. Second, the interpretation of selection functions is clarified to be the relative intensity of the Poisson process model, which can be used to quantify relative selection strength (Fieberg et al. 2021). This interpretation underlies the description of selection functions as a *relative* probability or intensity. If one were to fit the RSPF as described by Lele (2009), then an intercept is estimated and a different link function is used, such that one could in theory estimate the true probability of selection conditional on encountering a habitat unit (but, see caveats above and Hastie and Fithian 2013 for concerns with this approach). Third, the asymptotic equivalence between logistic regression and a Poisson point process indicates that, often, an extremely large number of available locations must be generated for the selection function to be accurately estimated using logistic regression (Warton and Shepherd 2010, Fithian and Hastie 2013, Northrup et al. 2013). Lastly, different modeling frameworks, such as Poisson regression, logistic regression or Maximum Entropy, all arrive at the same inference for habitat selection given an identical domain of availability and pixel size used in a GIS and, if using logistic regression, either a sufficiently large sample of available locations is taken, or infinite weighting (see Fithian and Hastie 2013) is used (Aarts et al. 2012, Muff et al. 2020). We note that the above information has often been repeated elsewhere. However, it is typically done in mathematical terms, which while more precise, lends confusion to those less versed in statistics. For the practitioner, the take home message is that, as long as the available distribution is sampled adequately (in terms of number of available locations) and that an exponential form for the selection function is assumed and used when making predictions, logistic regression is a perfectly suitable method for obtaining accurate and unbiased coefficient estimates for the selection function. Determining what constitutes an adequate sample, however, is not always straightforward, and often an exceedingly large sample of available locations is needed to approximate the weighted distribution (Warton and Shepherd 2010, Fithian and Hastie 2013, Northrup et al. 2013). Practically, analysts should conduct a sensitivity analysis to the availability sample size and assess the point at which coefficients converge to determine a sufficient sample (Northrup et al. 2013, Stabach et al. 2016, Fieberg et al. 2021). Alternatively, Fithian and Hastie (2013) show that by weighting the available locations, one can achieve similar results with fewer actual samples drawn. To do so using the *glm* function in R with argument “weights,” one would define a vector the length of the combined used and available sample and specify a 1 for each used location and specify a large number (e.g., 5,000) for each available sample.

## How Do Selection Functions Provide Inference to Habitat Selection?

**Fig. 3:**
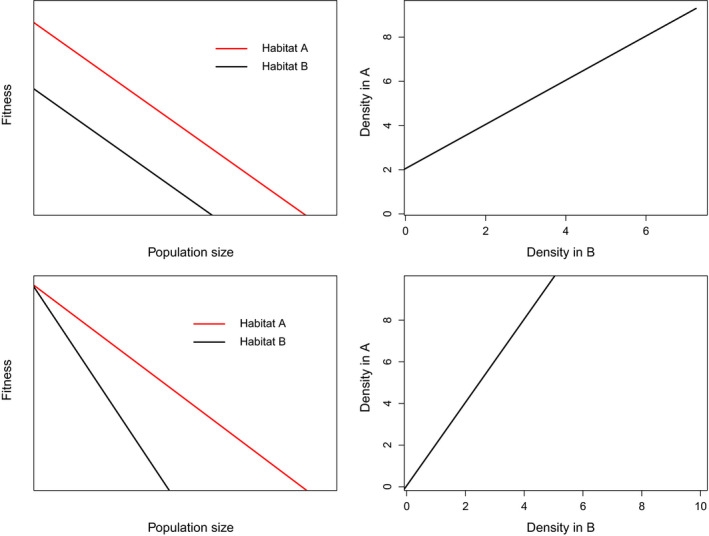
Two isodars (by row) illustrating simple scenarios under which differences in animal density can be found between habitats. In the first (row 1), the baseline fitness of animals (i.e., when density is 0) in habitat A is higher than in habitat B, but fitness changes equally in each habitat with density (slope = 1). In the second scenario (row 2), the initial fitness payoff is equal in the two habitats (bottom left panel), but declines more rapidly with animal density in habitat B.

For most researchers, HSAs are a tool used to obtain inference to the process of habitat selection, which is desired because of the well developed theoretical links between this process, fitness, and population distribution and regulation (Fretwell and Lucas 1969, Rosenzweig 1981, Morris 2003). However, rarely is the link between HSAs, as defined here, and habitat selection, as defined in the classical literature, explicitly made (but see van Beest et al. 2014*b*, Matthiopoulos et al. 2020). Habitat selection is variably defined in the literature, but generally can be thought of as the process through which individual animals differentially use or occupy available habitats (Morris 2003). Implicit in this definition is the discretization of habitats into distinct units. This discretization is important because it forms the basis of the Ideal Free Distribution (Fretwell and Lucas 1969), which is the foundation of habitat‐selection theory. This theory states that animals will select among discrete habitats such that their fitness is maximized at the time of selection, resulting in variation in population density among habitats in proportion to the value (in fitness terms) of each habitat. The Ideal Free Distribution considers habitat in coarse terms (two habitat types in the original work by Fretwell and Lucas) and a single, or few habitat‐selection decisions. In this early work, the authors used the example of birds “settling” in different habitats. However, the theory that forms the Ideal Free Distribution can be applied to continuous landscapes (Kshatriya and Cosner 2002) and over repeated choices among many habitats. That is, we can view the continual choices that animals make between different habitats as informed decisions about the fitness payoffs of those habitats relative to other available habitats at the time the decision is made and at the given population density. Faced with choices of habitats to select, those that are selected disproportionate to their availability will have a higher average population density. Because the Ideal Free Distribution is founded on the idea that at a stable state, mean fitness is equal among habitats, the ratio of densities between habitats provides information on the fitness payoffs of those habitats as perceived by the animal at the time of selection. Under the Ideal Free Distribution, animals are continually choosing which habitat to reside in based on the density‐fitness relationship. If population density declines in one habitat, such that per capita fitness is no longer equal among habitats, then animals will select the habitat with higher fitness until the per capita fitness once again balances out. Taking this view, we can make the inference that the habitat with higher density at any point in time is the habitat for which the average expected fitness *at the time of selection* was higher (although in practice there are many reasons to expect that density will not always reflect fitness; see Matthiopoulos et al. 2020 chapter 1). To test whether empirical patterns of animal distribution fit the Ideal Free Distribution, Morris (1987*b*, 1988, 2003) developed the isodar. In Box 1 and Fig. 3 we illustrate the utility of isodars for understanding the reasoning described above. Expanded to a more continuous context that is typical of HSAs, we can infer that habitats that are selected with higher intensity provided a higher perceived fitness payoff to the animal at the time of selection than alternatives and therefore will have a higher density over time.

Box 1 **Isodars and the Ideal Free Distribution.**

Isodars are a graphical representation of the Ideal Free Distribution, developed by Morris (1987*a*, 1988, 2003). Morris defines an isodar as “A line in the state‐space of habitat (usually) population densities where fitness…is equal in each habitat, but along which fitness varies.” Said another way, isodars are developed by plotting the density of animals in two discrete habitats against one another, such that the resulting line represents densities where the fitness of each animal in each habitat is equal. These lines represent an Ideal Free Distribution as predicted by Fretwell and Lucas (1969). Isodars can be highly complex (Morris 2003, 2011), but in basic linear isodars describing the original concepts of the Ideal Free Distribution, the pertinent information can be summarized by the intercept and slope. The intercept indicates the fitness payoff of each habitat for the first individual faced with choosing between two habitats. An intercept of 0, indicates identical fitness value of each habitat, while an intercept differing from 0 indicates that one habitat has a greater initial fitness payoff than the other. The magnitude of the intercept indicates how many individuals will need to occupy the habitat with the higher initial payoff before a single individual will select the other habitat. At this point, however, the fitness of individuals in each habitat is equal. The slope of the isodar indicates how fitness changes with animal density. A slope of 1 indicates that the change is equal across habitats and that new individuals settling in the habitats will do so sequentially alternating between the two available habitats. Slopes differing from 1 indicate a differential effect of animal density on fitness and can lead to multiple individuals settling in one habitat per individual settling in the other. In Fig. 3, we illustrate, with isodars, two potential scenarios under the Ideal Free Distribution that can lead to differences in animal density among habitats. First, the habitat with higher animal density could have a higher baseline fitness payoff. Second, density depresses fitness differently in each habitat. Either scenario leads to more individuals experiencing a higher fitness payoff at the time of selection in the habitat with higher density.

The above predictions are based on some assumptions, which we think are under appreciated in the habitat‐selection literature (but see Matthiopoulos et al. 2020 chapter 1). First, provided we assume that we can faithfully approximate the animal's perception of habitat with available environmental covariates, then we can assume that selection functions provide direct inference to these choices. Furthermore, assuming we have sampled animals long enough to obtain their steady‐state behavior, then we can assume that areas selected disproportionate to availability have a higher average density and therefore at the time of selection the higher expected fitness payoff for the selecting individual than alternatives. An additional assumption that must be made when making direct inference to the process of habitat selection in HSAs is that any environmental cues that an animal is using to select habitat are unaltered from the conditions under which the behavior evolved (Robertson et al. 2013). However, some examples might emerge whereby individuals may exhibit plasticity (Northrup et al. 2021) or species may adapt and track their habitat selection to match novel environmental cues. Meeting all the above assumptions might rarely occur (Matthiopoulos et al. 2020), particularly in human dominated systems. A further assumption implicit in the above line of reasoning is that habitat selection is density dependent, or that the marginal value of habitat decreases with increased competition (Morris 1987*a*). We discuss this point below (see Box 1 and *Density dependence*).

## Concepts and Advances

### The available distribution

The available distribution represents the distribution of habitat that the animal can select from. Implicit in this definition is the assumption that all habitat represented in the available distribution are accessible to the animal (Paton and Matthiopoulos 2016). This assumption has important implications for what the available distribution means in ecological and evolutionary terms, as accessibility is driven by an array of processes. These processes include an animal's perceptual range or cognitive map, physical constraints on distance moved, evolutionary history, social structure, and any other process that limits the ability of an animal to access a location at a given time (Garshelis 2000, Matthiopoulos 2003, Aarts et al. 2008, Beyer et al. 2010, Paton and Matthiopoulos 2016). Therefore, how these factors shape the available distribution should be considered in analyses and when making inference. Defining availability therefore plays an important role in several other concepts that we discuss below. For example, the definition of the available distribution is fundamental in assessments of scale dependence (Boyce 2006, Ciarniello et al. 2007, Northrup et al. 2016), functional responses in habitat selection (Mysterud and Ims 1998*b*, Godvik et al. 2009, Matthiopoulos et al. 2011), and how population density alters habitat selection (i.e., variation in density influences what habitat is accessible to an animal through intraspecific competition; Fretwell and Lucas 1969, Rosenzweig 1981, Morris 1987*a*, van Beest et al. 2014*a*). Therefore, ultimately, the choice of the available distribution will depend on the desired inference from the analysis, while at the same time constraining that inference. In other words, the results of any HSA provide inference on selection conditional on the definition of availability (Johnson 1980). This necessarily subjective nature of the available distribution (Beyer et al. 2010) also cannot simply be dismissed by appropriately stating that one's inference is conditional on availability; if the assumed available distribution contains large areas that were inaccessible to the animal, this can result in misleading inference and produce inaccurate predictions of habitat selection (Northrup et al. 2013, Paton and Matthiopoulos 2016).

There are many options for defining the available distribution. Often, these definitions are meant to capture one of the four orders of selection discussed by Johnson (1980). First order: selection of geographical space that covers the entire range of the species. Second order: selection of the home range of the individual in geographic space within the species' range. Third order: selection of habitat components within the home range. Fourth order: selection of food items from within a feeding site. Selection functions can be fit at scales between these orders, and we note that most examples in the literature fall somewhere between the second and fourth order. Most approaches in the literature have consisted of drawing availability uniformly from within different geographic extents, ranging from the study area to individual animal home ranges (Boyce 2006); these approaches are probably the most commonly used today. Earlier attempts to capture the fact that availability can depend on the location and time of a telemetry fix included drawing availability from within an area delineated by buffering used locations by biologically relevant distances, such as the mean distance between successful relocations (Boyce 2006). In such approaches, the used locations are matched with their location‐specific availability and selection functions are estimated using conditional logistic regression (e.g., Arthur et al. 1996, Duchesne et al. 2010) or Poisson regression with stratum‐specific intercepts (Muff et al. 2020). In these analyses, stratum indicates a set of used and corresponding available locations. More recently, researchers have attempted to define availability by considering animal movement constraints (see *Movement and HSAs*), allowing availability to vary dynamically over time according to a model of animal movement (Hooten et al. 2014, Avgar et al. 2016, Fieberg et al. 2021). Ultimately, these approaches are attempts to reduce the subjectivity of defining the available sample, but in reality, the researcher will never truly know what is available and accessible to the animal. Therefore, we suggest researchers conduct sensitivity analyses to the assumed scale of the available distribution (Northrup et al. 2013) or fit what are referred to as “generalized functional responses” that account for how inference changes with variation in availability (described by Matthiopoulos et al. 2011, Paton and Matthiopoulos 2016; see *Functional responses*). For analyses in which availability is matched to each used location, a relatively underutilized approach is to include covariates that directly relate to the accessibility of habitat. For example, including distance between the previous used location and the current used and proposed available locations will provide inference to how far an animal is willing to travel to access habitat. Including distance as a covariate in selection functions can also facilitate simultaneous estimation of movement and habitat‐selection processes by integrated step‐selection analyses (iSSAs; Avgar et al. 2016, Fieberg et al. 2021).

In general, we suggest that researchers interested in behavioral responses to environmental covariates over a short time period, or that have short intervals between telemetry fixes, should apply movement‐based availability sampling methods (but see *Scale dependence*). In addition, we suggest that researchers conduct sensitivity analyses using multiple definitions of the available distribution. Such an approach can highlight covariates for which there is a consistent response, indicating that they might be important drivers of the animal's behavior (Rettie and Messier 2000, Northrup et al. 2016). When prediction is the primary focus of an analysis, using generalized functional responses as described by Paton and Matthiopoulos (2016) can help strike an appropriate balance between bias and variance. Other methods, specifically developed for their predictive performance, such as machine‐learning algorithms and methods using different styles of statistical regularization (techniques that optimize the generalizability of a model; e.g., LASSO; Shoemaker et al. 2018, Gerber and Northrup 2020) also are available and likely underutilized in the HSA literature. Lastly, and most importantly, we strongly emphasize that all results must be interpreted in the context of the available distribution; all inferred selection behavior is conditional on the definition of availability.

### Statistical dependence in studies of habitat selection

Statistical dependence is a concern in most ecological studies. For HSAs, there are a suite of potential sources of statistical dependence arising from the repeat sampling of the behavior of individual animals (Fieberg et al. 2010) and temporal autocorrelation in an individual's locations and their associated environmental covariates, particularly when using short relocation intervals (Wittemyer et al. 2008, Boyce et al. 2010). When successive locations are close in space or share similar environmental covariates, it is either because the researcher has used a telemetry fix schedule that is at a finer temporal scale than the decision‐making process of the animal (see *Statistical dependence in studies of habitat selection*), autocorrelation in the landscape is so high that the animal has no variation to select from, or because the animal has made a decision to return to or stay in roughly the same area because of favorable habitat characteristics. In the first two cases, the analyst may need to consider how sample frequency and spatial autocorrelation of the landscape interact (Aarts et al. 2008), and then either rarify their data, model spatial autocorrelation arising from the sampling process (Johnson et al. 2013), or use a robust form of standard error that relaxes the assumption of independence (Nielsen et al. 2002, Fieberg et al. 2010). In the third case, we suggest that, because the use distribution is itself continuous and selection functions are providing inference to a process by which an animal selects from available habitat, every location, regardless of the presence of autocorrelation, provides information on this process. That is, every location is the result of selection, even in instances in which an animal remains stationary for long time periods. For instance, stationary behavior is the result of differential selection patterns depending on the behavioral state of the animal (e.g., the animal might be resting and therefore actively choosing to remain stationary). Another important consideration is the role that memory plays in determining if and when animals choose to return to past locations. Although habitat‐selection models incorporating memory are beginning to be developed (Merkle et al. 2014, Oliveira‐Santos et al. 2016), their application is rare. Therefore, although memory of locations will induce additional statistical dependence, understanding the subsequent influence on inference and how to address this influence are open questions (Van Moorter et al. 2013). The continued development of approaches that account for memory in habitat selection would lead to better integration of site fidelity theory into HSAs, which has implications for understanding ecological and evolutionary mechanisms of spatial and demographic processes (Gerber et al. 2019).

If an appropriate fix interval has been chosen, the decision to return to or stay at a location could be treated as an independent decision or data point (e.g., Lair 1987). In practice, however, the temporal scale used for data collection and modeling is often arbitrary, and we are unlikely to ever comprehensively quantify the drivers of selection. Furthermore, it is not clear how best to choose an appropriate available distribution when modern telemetry datasets often have fix intervals measured in minutes. Although some animals might be able to traverse their entire home range within the time between fixes, allowing for broader definitions of availability, in cases for which this is not feasible, the available distribution should be constrained at a location‐specific level (*Movement and HSAs*). In addition, it is inappropriate to maintain a consistent availability sample if there are uneven fix intervals within a telemetry dataset because, clearly, there is more area accessible to the animal over longer time periods. Ultimately, it is arguably most appropriate to constrain availability for each location based on the telemetry fix interval and the movement characteristics of the animal (Forester et al. 2009, Brost et al. 2015), but even under these approaches, decisions are often state dependent, and therefore, we often are quantifying the average selection behavior over an unbalanced set of states that will be unknown to the analyst. The suggested best practice of constraining availability by movement, however, is unsatisfying for those hoping to assess multiple orders of selection as outlined by Johnson (1980). For such studies, analysts will need to think about the appropriate use sample and scale of availability. If the full set of used GPS locations obtained from fine‐scale telemetry data are compared with, for example, study area or home‐range scale availability, there is likely to be dependence in the dataset and subsequent deflation of variance estimates that need to be accounted for (e.g., as outlined by Nielsen et al. 2002, Fieberg et al. 2010). Alternatively, when attempting to assess how individuals select home ranges from within the broader geographical range of the species, it is likely to be most appropriate to use an estimate of the home range itself as the use sample by quantifying environmental variables within the bounds of a home range estimator.

Repeat sampling of the selection behavior of individual animals is also known to induce statistical dependence when inference is desired at the population level (i.e., aggregated inference from multiple study animals). Furthermore, estimates of population‐level parameters may require properly accounting for unbalanced sample sizes across individuals. The continual advances being made in the area of hierarchical modeling (Gelman and Hill 2007), in which both individual and population‐level coefficients are estimated simultaneously (often referred to as random effects models) will largely make these issues irrelevant as the individual animal can more easily be treated as a unit of replication (e.g., Gillies et al. 2006, Hebblewhite and Merrill 2008, Fieberg et al. 2010, Northrup et al. 2015). We point readers to Muff et al. (2020) for a recent and important discussion of caveats needed when fitting hierarchical models in the context of HSAs, and to Schielzeth and Forstmeier (2009) for a more general discussion of the importance of considering random slopes (rather than the common practice of fitting models with only random intercepts). As a final point, we note that until recently, readily accessible and computationally efficient methods for fitting constrained availability models in a hierarchical framework—i.e., models that allow the animal, rather than the GPS location, to be treated as the unit of replication—were unavailable. However, Muff et al. (2020) described a computational trick for fitting these types of models efficiently using generalized linear mixed‐effects models. Although these models represent a major advancement in habitat‐selection modeling, allowing all coefficients in a model to vary by individual will lead to computational challenges.

### Context dependence in habitat selection

Behavioral tactics in animals are often context dependent (van Oers et al. 2005) or state dependent (McNamara and Houston 1996). Such dependency evolves when the tactic that maximizes fitness varies across circumstances (Dingemanse et al. 2010). These circumstances can include individual status, such as condition or rank (Gross 1996), or environmental context, such as the presence of conspecifics (van Oers et al. 2005). As with other behaviors, habitat selection is likely to be state or context dependent (McLoughlin et al. 2010), which offers both challenges and opportunities in HSAs. HSAs can elucidate state dependence, providing important insight to the underlying factors driving the evolution of habitat‐selection behaviors. However, the existence of dependence means that a failure to account for it can cause misleading inference; for example, we will often obtain results that combine different tactics or states (to which we are unaware) and therefore obscure important habitat‐selection patterns (McLoughlin et al. 2010).

McLoughlin et al. (2010) provide perhaps the landmark discussion of how dependency, in their terms: “ecological dynamics,” in HSAs can influence inference, focusing on how habitat selection can depend on population density, predation risk, and availability of habitat. Population density and the availability of habitat are two special cases of context dependence in HSAs. However, there is a range of other intrinsic and extrinsic factors that can cause dependence in HSAs, which have been addressed to varying degrees in the literature. Perhaps the most obvious form of dependence in HSAs is sex dependence, with habitat‐selection patterns expected to be different between males and females (Bouyer et al. 2015, Pigeon et al. 2016); see Box 2 for a discussion of technical aspects related to assessing factors such as sex dependence in HSAs. As McLoughlin et al. (2010) highlight, variation in predation risk can structure the way animals interact with their habitat, but this dependence extends to any species interaction including intraspecific (see *Density dependence* below) and interspecific competition (Stewart et al. 2002). Interestingly, although models have been developed for examining species interactions in hierarchical occupancy models (MacKenzie et al. 2004, Rota et al. 2016), and species distribution models (Pollock et al. 2014, Ovaskainen and Abrego 2020, Tikhonov et al. 2020), whereby the occurrence of multiple species are modeled simultaneously, with each depending, statistically, on the other, we are unaware of any similar treatments in HSAs using telemetry data. For social or territorial animals, it is likely that interactions with conspecifics are a dominant force driving habitat‐selection patterns (Moorcroft et al. 1999), but approaches for accounting for such dynamics are likely to be exceedingly difficult to implement (e.g., see Hooten et al. 2018 for an example in a different context), and difficulties are compounded by the fact that we never sample entire populations of animals. Seasonal dependence in habitat selection has been examined in some species (Nielsen et al. 2003, Mao et al. 2005, McLoughlin et al. 2011), although this topic is surprisingly less common than we would have expected. Many studies have assessed how habitat selection varies by time of day (Northrup et al. 2012*a*, Northrup et al. 2015, Dupke et al. 2017, Filla et al. 2017, Richter et al. 2020), assuming different pressures or different behaviors during these time periods, although what these are is not always clear (but see Pigeon et al. 2016, Street et al. 2016). Other forms of dependence that have been less frequently examined, but are likely to exist, include age dependence (e.g., senescence), condition dependence and dependence on reproductive status (Steyaert et al. 2013). If ignored, all of the above factors have the potential to provide misleading inference. Therefore, they should be addressed either by designing studies to explicitly capture any dependence or, if only population‐level inference is desired, ensuring a sufficiently large and representative sample of individuals to effectively capture the inherent variation that exists in unmeasured variables. In most systems, there are likely to be multiple sources of dependence, which could be intractable to sample across or disentangle, requiring controlled designs, e.g., only sampling females to account for sex dependence. Interestingly, in our review of the literature, many, if not most, studies address some form of dependence in habitat selection (e.g., time), but do not frame their results in this context, which serves to limit their impact.

Box 2 **Non‐spatial dependence and fitting selection functions.**

In our experience conducting HSAs and working with other researchers, we have encountered countless instances in which researchers want to assess factors such as sex or age dependence. Perhaps one of the most confusing aspects of conducting HSAs is that such forms of dependence, and indeed all forms of dependence that are non‐spatial, cannot be modeled simply by including an additive effect on the link scale. That is, one cannot simply include a covariate for sex or age in the model. To understand why, we remind the reader that HSAs typically are approximating a Poisson point process model, which is a spatial model, with an assumed underlying intensity surface. Including a non‐spatial covariate, such as sex, as an additive effect simply adjusts this surface up or down, depending on the direction and magnitude of the coefficient. i.e., a positive coefficient would just indicate that, for example, females (males) have an overall higher density (or proportion if using logistic regression) of used locations than males (females). However, these effects are entirely dependent on the number of individuals of each sex sampled, the number of telemetry locations obtained, and the number of available points sampled, for each animal. If, for example, by random chance, more telemetry devices malfunctioned on males than females, then we might have a greater number of location fixes on females, leading to spurious inference. Similarly, if the analyst were simply to generate more available locations for one sex over the other, similar spurious inference could occur. Therefore, for researchers aiming to assess non‐spatial dependence, including sex, age, seasonal, temporal, or behavioral dependence, separate models for each group should be fit, or interactions should be included between every covariate of interest and the non‐spatial covariate (e.g., sex × elevation), such that inference can be made to how the different groups select specific resource types. Alternatively, as discussed by Erickson et al. (2014), one can model the *difference* in selection between two groups by comparing the used locations of the two groups in a logistic regression (coding used locations from one group as 1s and the other as 0s). This modeling framework requires an assumption of equal availability between the two groups to be valid.

An exciting and emerging area of research involving dependence in HSAs is the explicit incorporation of behavioral states. Animals engage in many different activities throughout the day, such as foraging, resting and mating, and the resources they require for these activities are likely to vary. Ignoring behavioral dependence in habitat selection can strongly impact inference (Roever et al. 2014, Zeller et al. 2014, Abrahms et al. 2016), but addressing it is not entirely straightforward as it requires the delineation of behaviors. Although methods exist for such delineation, including intensive field investigations to validate behaviors (Wilson et al. 2012, Bouyer et al. 2015), or the use of auxiliary sensors (Abrahms et al. 2016), they require some degree of field‐intensive validation. Advances in statistical movement modeling provide a range of methods for categorizing location data into different putative behavioral states (see Gurarie et al. 2016). These methods provide a clear path toward assessing behavior‐dependent habitat selection and are beginning to be applied in this literature (e.g., Roever et al. 2014, Zeller et al. 2014). Although statistical delineation of behaviors provides a desirable approach for researchers, there is uncertainty in the state delineation, suggesting that the state delineation and selection function should be fit simultaneously, as highlighted by Nicosia et al. (2017). The benefits of addressing behavioral dependence are immense, because animals are likely to select disparate resources for different behaviors; an ungulate might require dense brush for resting habitat, but open grasslands for foraging habitat. Behavior‐specific analyses can also reveal the ways in which animals acclimate to modified landscapes (Bouyer et al. 2015). Therefore, differentiating these habitat types is critical for understanding how best to manage and conserve species. However, identifying behavioral dependence raises additional challenges, as once defined, researchers might need to determine which behavior is more important for the management of the species, or which behavior‐specific habitat is more limiting. These areas are open and active questions in the field.

### Scale dependence

Levin (1992), in his foundational treatise on scale noted that “relating phenomena across scales is the central problem in biology and in all of science.” The scale at which a study is conducted has profound implications on our understanding of an array of processes (Whittaker and Lindzey 2004, Laforge et al. 2015*a*), including movement (Johnson et al. 2002) and habitat selection (Ciarniello et al. 2007, Mayor et al. 2009, McGarigal et al. 2016, Bastille‐Rousseau et al. 2017). Differences in ecological processes across scales ultimately stem from the notion that decisions by animals on where to spend time occur hierarchically, with slower processes, such as populations selecting appropriate landscapes, occurring over longer periods of time than more immediate decisions (e.g., individuals deciding on a local patch within which to forage; Rettie and Messier 2000, Mayor et al. 2009). Furthermore, processes acting at one scale can influence and even give rise to patterns and processes at broader spatiotemporal scales (Van Moorter et al. 2016). Therefore, how scale is defined in HSAs has strong implications for inference.

Scale is variably defined in the literature, but here we refer to scale relative to its three components: extent, grain and resolution. Extent is the complete areal or temporal coverage of an analysis, grain is the area or time period surrounding a point or observation over which environmental variables are considered (Anderson et al. 2005, Meyer and Thuiller 2006), and resolution is the minimum mapping (when discrete space is considered) or temporal unit of data. Although grain and resolution are similar, and can be identical, grain is entirely under the control of the analyst and can be calculated in continuous space (e.g., the number of houses with a 50 m radius around used and available points) while resolution is strictly in discrete space and is often simply the minimum pixel size in a remotely sensed raster. All three components of scale can be under control of the researcher and are fundamental to any HSA, having both practical—i.e., are fundamental to how an analysis is actually carried out—and inferential implications. Varying any of the components of scale can offer different insight and inference into ecological process. However, the three components are variably discussed in the HSA literature. Extent, in a spatial context, is the component of scale that is most often explicitly treated in HSAs (McGarigal et al. 2016). This explicit treatment is likely because extent is typically tied closely to the available distribution, with researchers often choosing to make the extent of inference and availability identical. This choice is made because selection functions are typically only predictive in the areas where they were fit due to differences in the relative abundance of habitat in other areas, missing predictors or model misspecification that will result in coefficients that poorly reflect causal effects (Matthiopoulos et al. 2011, Fourcade et al. 2018; see *Functional responses* for a discussion of functional responses). Furthermore, researchers have often aimed to vary the spatial extent of availability to assess the factors influencing habitat selection at different extents (Boyce 2006), typically chosen based on the Johnson (1980) orders of selection. When investigating habitat selection across different extents, studies may simply opt to generate separate models at more than one scale and qualitatively compare results (Rettie and Messier 2000, Ciarniello et al. 2007). However, techniques have been developed to integrate selection functions across multiple extents into a single model. For example, Johnson et al. (2004) multiplied relative probabilities of selection predicted from models fit across multiple spatial extents to produce a final map. To account for nestedness across selection orders, DeCesare (2012) used a method which integrated selection orders as the product of conditional relative selection at each scale into a single quantity. Bastille‐Rousseau et al. (2017) showed that by incorporating decision‐making processes acting at multiple spatial extents into HSAs, one can improve predictions, while Zeller et al. (2014) showed that the estimation of landscape resistance can be improved by taking a multiscale approach. Furthermore, Van Moorter et al. (2016) demonstrated that habitat‐selection patterns scale upwards to emergent properties of space use at broader spatiotemporal scales.

Simulation and sensitivity analyses have demonstrated that mismatch between the extent of the data generating process and analysis can lead to bias in HSAs (Northrup et al. 2013), highlighting the importance of correctly specifying spatial extent. Choice of scale is also bounded on one end by the extent of data collected, and on the other by the spatial and temporal resolution of both use data and environmental variables. One cannot make inferences on the geographic range of a species from data collected in a single study area, and making inferences about forage selection for herbivores is impossible without very fine‐scale information on plant composition, which is typically not available using remote sensing technologies. Although extent is most commonly conceived of in terms of space, time is also an implicit component of extent in HSAs. Clearly, results are only directly applicable to the time period over which data were collected, but more fundamentally, if the movements of animals are used to define home ranges over which availability is drawn, or if more complex, movement‐based definitions of availability are used (Hooten et al. 2014, Avgar et al. 2016, Northrup et al. 2016), then temporal scale becomes a more fundamental component of the analysis, influencing both use and availability.

Resolution and grain are often reported in habitat‐selection studies, but their influence on results is less often explicitly examined (McGarigal et al. 2016). Grain is an often overlooked but critical component of HSAs. If approached appropriately, it provides direct inference to how an animal perceives its environment. The concept of grain in this context is similar to the concept of the “zone of influence” around ecological disturbance (Boulanger et al. 2012), as both describe a certain threshold distance over which a resource or disturbance affects animal habitat selection and can vary based on context (Kite et al. 2016). Meyer and Thuiller (2006) recommended constraining the grain of analysis to the next finer level of analysis in the hierarchy of the process being examined (e.g., for a landscape‐scale analysis, grain should be the size of individual home ranges) to avoid conflating selection across domains of habitat selection. However, such an approach does not allow for investigation over multiple grain sizes (Anderson et al. 2005, Leblond et al. 2011), which can improve performance of models (Meyer and Thuiller 2006). Laforge et al. (2015*b*) recommended the use of different grains for each habitat covariate and proposed a two‐step method for inferring the most relevant grain size by comparing across grains using Akaike's Information Criterion (AIC). We recommend care be taken to minimize the number of models fit to avoid overfitting of the data due to the large number of models fit (Fieberg and Johnson 2015). Other studies have constructed “multilevel” models by first optimizing the chosen grain and integrating selection functions fit at different orders of selection to generate a single predictive multiscale model (Zeller et al. 2017, Fattebert et al. 2018). Paton and Matthiopoulos (2016) show that by incorporating habitat covariates measured at multiple grains into a single analysis, one can improve transferability of models to new systems. McGarigal et al. (2016) argue that such an optimization is critical for ensuring appropriate inference in habitat‐selection studies. Although this is an area of open research, we generally suggest that researchers aim to define grain based on the biology of their study system and avoid data‐driven modeling strategies; this is also a good strategy for avoiding overfitting.

Resolution defines the minimum pixel size of raster data (although some authors refer to this as grain) and is the least examined aspect of scale in the HSA literature. We do not discuss spatial resolution here as it often will be dependent on available spatial covariate data. However, temporal resolution (i.e., the fix interval) is crucial to insights in HSAs, as fundamentally different behavioral patterns are being sampled across different time scales (Northrup et al. 2016). This area has not received much attention in the literature.

How one addresses scale in an HSA is paramount to appropriate inference and depends on data and technological limitations, as well as what type of inference the analyst desires. Spatial and temporal resolution of data are often based on the limitations of technology (e.g., remote sensing data and GPS telemetry collars). In cases in which prediction is the primary intent of an analysis, extent is somewhat easier to resolve as one can choose the area over which they desire predictions and a model fitting algorithm to maximize predictions (such algorithms could easily incorporate covariates measured over multiple grains). When ecological or evolutionary insight is desired, it is less clear that researchers can simply choose one extent or grain (see McGarigal et al. 2016) to focus on and be confident that their results are robust to multiscale processes. When possible, we advise using methods that explicitly incorporate multiple extents, or conduct analyses across extents to compare how responses to environmental factors change. We further suggest that at least one of these extents be defined by the movements of the animal. The same general advice holds for grain, for which substantial insight can be gained by varying the area over which environmental features are quantified to identify the optimal grain, potentially indicative of the scale at which the animal perceives the environment, although, as above, we caution against potential overfitting that could occur if the number of models is not limited in some way.

### Functional responses

**Fig. 4:**
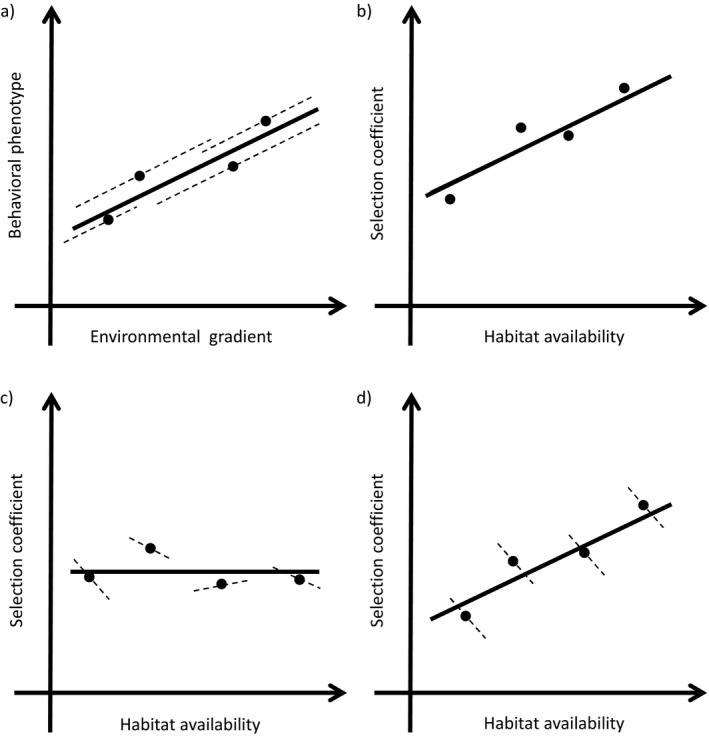
Similarities can be drawn between behavioral reaction norms (a) and functional responses in habitat selection (b). Functional responses in habitat selection (b) are often evaluated with an interaction term between an environmental covariate and its availability (see *Functional responses*) which informs us about the population‐level response (thick line) based on the mean individual selection coefficients (dots). Two‐step approaches or hierarchical modeling can allow us to determine intraindividual variation in behavior (i.e. behavior measured on one individual across different habitat availabilities, dotted line) and the population‐level response (thick line). This distinction between intraindividual and interindividual variation is important because patterns found at the population level might not hold true at the individual level, or *vice versa* (c, d).

An important form of dependence in habitat selection comes from the fact that this process can vary strongly with environmental context. This dependence is captured by fitting what are referred to as functional responses, which measure the change in the magnitude of selection for a resource type with changing availability (Fig. 4). The concept of variation in behavior across differential resource availability comes from the classic work by Holling (1959a, 1959b) who discussed functional responses as the change in prey capture rates with changing prey density. Functional response terminology was first adapted to habitat‐selection studies by Mysterud and Ims (1998*a*) and captures the idea that an animal's selection of habitat depends on the availability of all habitat in the landscape (Godvik et al. 2009, Beyer et al. 2010). Although interpretation of functional responses can be complicated by the fact that they can arise from different underlying behavioral processes (Beyer et al. 2010, Holbrook et al. 2019), examining them still can provide important insight to trade‐offs in habitat selection that animals undertake to meet demands and maximize fitness (e.g., food vs shelter; Mauritzen et al. 2003, Mabille et al. 2012). Furthermore, functional responses can elucidate potential links between habitat selection and population dynamics (Matthiopoulos et al. 2015, Matthiopoulos et al. 2019). Habitats each have intrinsic values, but the costs and benefits of using habitat may depend on its availability or changes in the availability of other habitats. For instance, a closed‐canopy coniferous forest may act as a refuge more effectively (or only) when available in large tracts. Similarly, the relative use of a food resource by prey may be higher if there are refuges from predators nearby. Therefore, functional responses may shape coefficient estimates in selection functions and may lead to incorrect inferences and poor predictions if not considered (Aarts et al. 2013, Paton and Matthiopoulos 2016).

Functional responses can be assessed by examining the relationship between the availability of an environmental covariate (often the average availability over some predetermined spatial extent) and estimated coefficients in a selection function. Matthiopoulos et al. (2011) described a method whereby functional responses are estimated conjointly with selection function coefficients through an interaction term between a resource covariate and its availability (see also; Godvik et al. 2009, Aarts et al. 2013, Leclerc et al. 2014, van Beest et al. 2016). More commonly, analysts work in two stages. First, they fit either individual models or population‐level hierarchical models with coefficients (β) varying by individual animal (i.e., a model with slopes varying by individual; Hebblewhite and Merrill 2008, Mabille et al. 2012), followed by a separate regression on the relationship between coefficients and availability. Commonly, researchers will simply use the mean resource availability and the point estimates (β coefficient) from the selection functions (e.g., Northrup et al. 2016), which fail to appropriately propagate parameter uncertainty, leading to inappropriate variance in the functional response (Hadfield et al. 2010, Houslay and Wilson 2017). We note, however, that methods have recently been developed for two‐stage Bayesian analyses that could appropriately propagate uncertainty between the individual β coefficients and the resource availability in the functional response model (Lunn et al. 2013, Hooten et al. 2016). However, general patterns should emerge at the population level regardless of the approach taken (see van Beest et al. 2016). Contrastingly, although hierarchical models are typically preferred to individual models, if functional responses are present, but ignored in the first step of modeling, the hierarchical model (individuals as samples from a general population) approach could lead to underestimation of the functional response due to the shrinkage of individual coefficients toward the population mean. Therefore, if functional responses are of fundamental interest, then they should be fitted in a single step as part of a hierarchical model (individuals as samples for a functional response).

Recent studies are not only trying to document patterns of functional responses in habitat selection, but also to understand how they vary in time or space. Indeed, different functional responses can be observed depending on scale (Laforge et al. 2016, Northrup et al. 2016), season (Mauritzen et al. 2003, Godvik et al. 2009) and population density (van Beest et al. 2016). Such results can help us better understand functional responses in habitat selection and their ecological and evolutionary consequences (Leclerc et al. 2014, Losier et al. 2015). Despite these advances, there are still several lines of research that have been under‐investigated. Although population‐level functional responses have provided important insight to habitat selection, one could examine selection function coefficients over time or an environmental gradient for a single individual and gain detailed insight on the trade‐offs animals make across gradients of habitat availability at the individual level. Indeed, with growing capabilities to track animals across multiple years, there is an incredible opportunity to assess functional responses at the individual level. Such an approach would be akin to a reaction norm, as outlined in Fig. 4 and discussed by Dingemanse et al. (2010). Furthermore, most studies of functional responses examine how an animal's response to a single environmental variable varies as a function of the availability of that variable (e.g., how selection coefficients for forest cover vary over different proportions of the landscape comprised of forest). However, selection probably varies as a complex function of the availability of multiple environmental variables (Matthiopoulos et al. 2011). Lastly, how the scale of habitat availability is assessed, in the second stage of a functional response, and how this influences inference from functional responses is an area that is understudied. Functional responses probably exist at multiple scales (Beyer et al. 2010, Northrup et al. 2016), but how habitat selection at one scale varies with availability across scales is an area of open research.

### Density dependence

The foundations of habitat‐selection theory are built on the concept of this process being density dependent (Fretwell and Lucas 1969). Animal habitat‐selection decisions cannot be separated from the context of population density under which they took place. As such, intraspecies competition is central to all HSAs. Among the most important contributions of McLoughlin et al. (2010) was the conceptual link between the foundational principles of density‐dependent habitat selection and contemporary HSAs. The authors synthesized concepts from the ideal free and ideal despotic distributions (Fretwell and Lucas 1969), isoleg (Rosenzweig 1981), and isodar (Morris 1987*a*, 2003) analyses, providing predictions of how animals distribute themselves across coarse‐grained habitats as a function of the relative profitability, i.e., fitness, of a habitat (see Box 1). Density‐dependent habitat selection predicts that, as population density increases, on average, populations will generalize their habitat choices (Fortin et al. 2008). Although a concept originally intended to apply to a coarse‐grained HSA (i.e., with two habitats only), this prediction bridged these more simplified models of selection, and became applicable to multiple‐covariate, fine‐grained HSAs. For example, van Beest et al. (2014*a*) empirically bridged the divide from classical density‐dependent habitat‐selection theory to HSAs, demonstrating that, as population density increases, selection becomes less pronounced for certain habitats (see also Huntsman et al. 2017, Robson and van Aarde 2017).

Central to the challenge of integrating population density into HSAs, noted early by Boyce and McDonald (1999) and again by Boyce et al. (2016), is that density‐dependent habitat selection and its relationship to HSAs suggests that most analyses are snapshots in time that probably only pertain to the population density during the time of sampling (Avgar et al. 2020). McLoughlin et al. (2010) offered some clear practical guidance for including density when fitting selection functions, however few studies published since 2010 have addressed this topic. Those studies that have addressed population density, have done so primarily in three ways. First, density is integrated explicitly into the selection model as a covariate that interacts with habitat (van Beest et al. 2014*a*, *b*, Sollmann et al. 2016, van Beest et al. 2016, Robson and van Aarde 2017). Second, density is viewed generally as an outcome whereby authors try to understand how population size, or carrying capacity can be predicted by population‐level selection (Robinson 2015, Street et al. 2017). Lastly, density is acknowledged to influence selection (Meisner et al. 2014, Pietrek and González‐Roglich 2015, Stewart et al. 2015) but not integrated empirically.

We recognize there are methodological and biological challenges when dealing with density‐dependent processes within an HSA. Interannual variation in population density may be useful for studies that include multiyear information of an animal's space use, whereby density in a given year can interact with selection within the same year. For large vertebrates, general estimates of density may exist (Santini et al. 2018). However, error around annual population estimates often exceeds any annual variation in population size. Moreover, it is difficult to disentangle the influence of annual density from other “year” effects, primarily annual variation in forage or other important resources. Furthermore, for species in highly temporally variable environments, effective population density and environmental factors such as forage availability might be closely linked. Conversely, capturing population density at a finer‐resolution (e.g., group size Fortin et al. 2009) permits a better understanding of the fine‐scale variation in selection with density and can be incorporated as an interactive effect in selection functions (Box 2, McLoughlin et al. 2010). We recommend that, at minimum, authors need to acknowledge in their studies that their results are specific to the population size at the time of study.

### Movement and HSAs

Movement (i.e., the displacement of animals in space over time) is the mechanism underlying an array of ecological and evolutionary processes (Nathan et al. 2008). Animals move to acquire resources (Owen‐Smith et al. 2010), precipitate or diminish interactions with other animals, and ultimately facilitate how they distribute themselves on the landscape (Turchin 1991, 1998). Subsequently, these processes are linked to nutrient transfer, the maintenance of genetic diversity, and spatiotemporal patterns of biodiversity (Jeltsch 2013). Indeed, movement is a component of nearly every ecological and evolutionary process.

Movement and habitat selection are tightly intertwined (Van Moorter et al. 2016). Habitat affects an animal's movement patterns, movement is the process by which animals select habitat and the capacity for movement directly affects what is accessible to an animal (Matthiopoulos 2003, Avgar et al. 2016, Spiegel et al. 2017). The data used in HSAs are subsequent locations of animals in space and time (most commonly GPS radio collar data) and, as such, are samples of the movement process. This fact raises two important analytical issues. First, the data used in HSAs are autocorrelated in space and time (see *Statistical dependence in studies of habitat selection*). Second, the sampling resolution of animal movement (i.e., the telemetry fix interval) influences what is accessible. Therefore, movement is a fundamental component of any HSA, whether it is explicitly or implicitly treated.

Numerous approaches have been developed to attempt to account for the process of movement in HSAs. Here, we present some of the methods that have evolved over the past two decades. First, one can fit a traditional selection function (i.e., an RSF), in which all locations from an individual are treated identically with a static sample of availability drawn from an area such as the bounding polygon from a home‐range estimator or a study area extent. These methods might incorporate movement, such as when fitting a home‐range estimator (Fleming et al. 2015), or using movement data to first delineate putative behavioral states prior to fitting selection functions (Roever et al. 2014). Several methods exist for segmenting movement data including hidden Markov models (Morales et al. 2004) and behavioral change point analysis (Gurarie et al. 2009). Similar to other forms of non‐spatial dependence (Box 2), once states have been identified, a separate selection function would need to be fit to data from each state to make inference to habitat selection. However, there are considerable opportunities to incorporate movement into HSAs that are not fully realized in this static and simplified approach. The second approach is to use observed movement behavior to bound what is available to the animal in a conditional selection function (e.g., an SSF). In this approach, each used location is matched with a set of available locations based on spatial or temporal ranges (Arthur et al. 1996, Compton et al. 2002, Boyce et al. 2003). For example, Arthur et al. (1996) used a standard buffer to define the extent of location‐specific availability, with the buffer extent defined as an animal's estimated movement distance between observations. Indeed, there are numerous methods described in the literature for defining the available domain at each used location and the majority rely on the movement of the animal to do so.

The method termed the SSF (*sensu* Fortin et al. 2005) and reviewed by Thurfjell et al. (2014), and more recently by Fieberg et al. (2021) allows for non‐uniform availability at each location and can actually provide inference to the factors influencing the movement of animals by using covariates that reflect environmental conditions on the path connecting sequential locations. This approach is a more informed consideration of movement in the context of availability, and accounting for animal movement in HSAs reduces bias in inferences (Forester et al. 2009). SSFs as first described by Fortin et al. (2005) incorporate variability in movement rates by drawing the available locations randomly using an empirical distribution of observed step lengths and turn angles. Hooten et al. (2014) expand on this approach and present a continuous formulation of both the used and available distributions by using a continuous time correlated random walk model. Step‐selection functions can be fit with standard software for fitting conditional logistic regression models, which makes them appealing to researchers. However, while hierarchical (or random effects) models for RSFs have been tractable in standard software for over a decade, until recently, it was difficult to fit SSFs in a computationally efficient manner while also accounting for the hierarchical structure of data collected on multiple animals. Muff et al. (2020) outlined a solution to this issue by using a Poisson formulation of the conditional logistic regression model.

When fitting SSFs, the model is formulated using a selection‐free movement kernel that describes how the animal would move in the absence of habitat selection, multiplied by a habitat‐selection kernel that describes the relative attractiveness of different areas on the landscape. However, movement and habitat are non‐independent processes. To address this issue, Avgar et al. (2016), expanding on work by Forester et al. (2009) and others, described an integrated step‐selection analysis (iSSA) which formulates the selection‐free movement kernel in terms of step length and turn angle distributions; this movement kernel determines the available distribution associated with each observed location. During the modeling phase, parameters in both the movement and selection kernels can be simultaneously estimated. In addition, it is possible to allow the movement kernel to depend on the habitat by including interactions between movement characteristics (e.g., step length, cosine of the turn angle) and environmental predictors measured at the previously observed location (Avgar et al. 2016, Prokopenko et al. 2017). Fieberg et al. (2021) provide a “how to” guide for conducting iSSAs using the amt package in R (Signer et al. 2019). Importantly, the iSSA approach produces an empirically parameterized mechanistic movement model capable of translating fine‐scale movements and habitat‐selection behaviors to coarser scale distributions (Potts et al. 2014*b*, Avgar et al. 2016, Signer et al. 2017).

An HSA framework that incorporates animal movement can aid in understanding the ecology of a system in addition to reducing bias in habitat‐selection inferences. The most appropriate means of fitting selection functions will depend on the desired inference, as is the case with other practitioner decisions. If ecological understanding, as opposed to prediction, is desired, we suggest that movement should be incorporated into analyses using the SSF framework because the constraints imposed on availability make conceptual sense and are statistically more robust for modern telemetry data, which are serially autocorrelated. When forming available distributions, we further suggest that researchers use a parametric approach, using common statistical distributions to model step length and turn‐angle distributions, as opposed to resampling from the empirical distributions of turn angle and step length (Forester et al. 2009), although we note that to date there has been no assessment of the bias introduced by choosing one of these methods over the other. If inference is also desired on the actual movements of the animal, the approach of Avgar et al. (2016) should be considered. We do caution, however, that when applied in a hierarchical modeling framework, that Muff et al. (2020) found that this approach, which typically entails including step length as a covariate in models, led to biased estimators of variance parameters. The reason for this bias is unclear, and resolving this issue is an important area of ongoing research. Lastly, the field of movement ecology has developed a plethora of methods for examining the causes and consequences of movement itself. This area of research is fast evolving and a fulsome treatment of the links between movement and habitat selection, or even of the SSF and iSSA literature is beyond the scope of this review. New advances that directly incorporate movement into analyses of habitat selection are already being developed with many more on the horizon, and we anticipate that these approaches will provide interesting pathways for simultaneous inference to these related process (e.g., Hooten et al. 2010, Hanks et al. 2015).

### Individual variability

There is an established link between individual differences in behavior and broader ecological and evolutionary patterns (Wolf and Weissing 2012). Phenotypic variation is required for natural selection to act upon and animal behavior can be treated similarly to other phenotypes (Duckworth 2009). Individuals in a population can display variability in a myriad of behavioral characteristics (Bell et al. 2009), including individual differences in habitat selection (Leclerc et al. 2016, Hertel et al. 2019). These differences present both a challenge and an opportunity in HSAs.

Individual variation is a theme that runs throughout most of the other topics addressed in this review (e.g., availability, functional response, density dependence) but is a critical component to consider when assessing habitat selection, and as such, we provide a limited discussion here. To date, much of the work involving variation among individuals in HSAs has focused on either methods for dealing with statistical dependence introduced by repeated sampling of individuals or on using individual variation to understand the functional response, i.e., how environmental factors influence individual variation in habitat‐selection behavior. However, individual differences in habitat selection can arise from a multitude of factors outside of plastic responses to environmental variation, including natal experience (e.g., Silver Spoon Effects; Stamps 2006, Stamps et al. 2009), the existence of different behavioral syndromes within a population (Sih et al. 2004), intraspecific and interspecific interactions (Fletcher and Miller 2006, Svanback and Bolnick 2007) and physiology (Biro and Stamps 2010). Therefore, there is much to be learned by treating individual variation as a feature to be explored, rather than a nuisance. Considerable effort has been devoted to understanding individual differences in the field of animal behavior, yet ecological studies on habitat selection have largely ignored these differences. In future work, we encourage attempts to incorporate what has been learned from animal behavior into analyses of habitat selection (e.g., Wittemyer et al. 2019). Echoing a common refrain from previous sections, how researchers account for or leverage individual variation in habitat‐selection behavior depends largely on the goal of the study. The consideration of individual variability in habitat selection improves our population‐level inferences and predictions and the variation around them, but these endeavors become increasingly valuable when the causes and consequences of this variation are tested (e.g., Montgomery et al. 2018, Bastille‐Rousseau and Wittemyer 2019).

From a technical perspective, we suggest that researchers quantify individual variability and incorporate the uncertainty this variability introduces into population‐level estimates of habitat selection using hierarchical models as outlined by Muff et al. (2020). However, these models are more technically challenging to fit. Therefore, an alternative approach is to fit separate selection functions to each individual animal, which allows for an understanding of how much variability exists within the sampled population. As a further step, analysts could then treat the estimated coefficients from individual selection functions as “data” in secondary analyses to explore potential underlying reasons for the variability (e.g., Murtaugh 2007). When predictions are of interest, it is less clear, from the literature, what is the most effective approach to accounting for and incorporating individual variability, and indeed this is an area of much needed research. Because of the log link used to model the data, predictions formed using averaged coefficients will differ from those obtained by averaging predictions from the individual models, with the latter approach being more appropriate for characterizing population‐level patterns (Fieberg et al. 2009).

### Uncertainty, mapping and inference

Uncertainty is inherent to any study involving sampling and statistical model fitting and needs to be appropriately quantified to evaluate the utility of a model and make appropriate ecological inference (Hooten et al. 2017). As with any ecological model, HSAs involve some factors that generate error, variability, and uncertainty. Measurement error is generated by fixes from telemetry devices that observe the location of the animal imperfectly (Frair et al. 2004, 2010). The characteristics and magnitude of this location error will depend on the technology, and methods have been developed to incorporate this uncertainty into parameter estimates (Brost et al. 2015, Gerber et al. 2018). Habitat‐induced bias in fix success is common and introduces further error that can be addressed by explicitly modeling the probability of a successful fix (Hebblewhite et al. 2007, Nielson et al. 2009) or integrating HSAs with animal movement models (Brost et al. 2015). Other methods for dealing with measurement error include censoring locations that are likely to have the highest degree of uncertainty (D'Eon et al. 2002, Lewis et al. 2007), although this approach leads to the loss of potentially valuable data and could lead to dropping data non‐randomly if poor precision locations occur more often in certain habitats. Model uncertainty can be assessed by model comparison or can be addressed using model averaging through information‐theoretic criteria. Furthermore, one can perform qualitative sensitivity analyses to assess the robustness of conclusions to different model assumptions. When prediction is the focus, there are many approaches to conducting optimal predictive model fitting including machine‐learning algorithms such as MaxEnt, boosted regression trees and random forests (Elith and Leathwick 2009*b*), or parametric approaches such as LASSO (Gerber and Northrup 2020); we suggest researchers use these approaches for prediction.

As with any analysis, sampling variability induces uncertainty in parameter estimates (e.g., as quantified by the standard error of a coefficient). The primary source of sampling variability in HSAs comes from among‐individual variability, but also from the fact that each individual is only sampled for a portion of their lifetime. This can lead to uncertainty in individual‐level coefficients as well as population‐level parameters (i.e., means and variances of individual coefficients). Assuming a random sample of individuals, we may have confidence that our population‐level inference applies to the true population of animals. However, this variation must be accounted for explicitly in the modeling framework either through fitting hierarchical models, or individual models, followed by secondary data analysis (see *Individual variability* for more detailed discussion of incorporating individual variability into selection functions). Lastly, the scale of availability (*The available distribution*) is a fundamental component of HSAs, determining the type of inference being sought. Considering multiple availability scales might be needed to gain a clear understating of habitat selection (Northrup et al. 2016, Paton and Matthiopoulos 2016, Michelot et al. 2019).

An additional source of error in HSAs that is rarely addressed is the uncertainty in the underlying spatial covariates used to infer patterns of habitat selection. Researchers often assess selection of habitat using remotely sensed products (e.g., the National Landcover Database in the USA). These products have error associated with them (Wickham et al. 2017), which introduces error to HSA results and any map that is produced. There is further error introduced from the discretization of spatial layers and therefore the averaging of information across pixels. To date there has been little assessment of the impacts these issues have on HSA inference. In our opinion, most remotely sensed spatial covariates represent, at best, a proxy for true ecological processes and researchers should always have a clear *a priori* hypothesis regarding the biological and ecological processes relating to any spatial covariate. We do note that HSAs are commonly used to assess the influence of human disturbance (Northrup et al. 2015). Human‐created features tend to have hard edges that are easier to characterize from satellite imagery. Therefore, there might be less uncertainty associated with spatial covariate data related to human disturbance, although this should not excuse researchers from developing clear hypotheses at the outset of these HSAs.

Habitat‐selection studies routinely map the relative selection strength as a means to visualize the behavioral patterns of the animal. Such maps are often provided to resource managers for use in decision making and conservation planning. In providing maps of selection functions, researchers need to convey the uncertainty in their mapped estimates. Furthermore, maps of relative selection strength commonly depict mean predictions, which do not convey the degree of uncertainty in estimated coefficients. We recommend each mean‐selection function map be accompanied by an equivalent set of uncertainty maps, displaying the range of predictions (minimum to maximum) or the coefficient of variation. Perhaps an even more critical issue is that of conveying the scale of availability used for the estimated selection parameters. Habitat‐selection predictions from a used/available design are not the probability of habitat use (Lele et al. 2013, MacKenzie et al. 2017), which is a natural interpretation and one easily misunderstood when researchers analyze their data using logistic regression. Given that a spatial location is predicted to have a high relative selection strength, its interpretation should be based on the environmental features at that location, given all the features that would be available and accessible to an animal. Therefore, interpretation of a selection function map is misleading without considering the habitat available to an animal, which may vary both spatially and temporally (Michelot et al. 2019). Depicting availability and selection at a spatial location is conceptually challenging, particularly when researchers are interested in fitting models that directly incorporate movement, such as SSFs. For any selection function, the specific interpretation of a mapped pixel is the relative use of the pixel conditional on that pixel being available to the animal. For an analysis with constant availability, the conditional part of the previous sentence can be dropped, leaving us with the definition of the relative use of a set of pixels, or an estimate of the utilization distribution. For conditional selection functions with availability constrained by movement (i.e., SSFs), availability is obviously not constant, so the simple plots of the selection function (i.e., $\exp\left(x\beta \right)$) are less meaningful. For these conditional selection functions, an estimate of the utilization distribution is still achievable but requires simulation (Signer et al. 2017) or solving for the steady‐state distribution of the underlying movement model (Potts et al. 2014*a*). Michelot et al. (2019) discussed promising advances in reconciling predictions across behavioral scales, and we look forward to future advances on this topic. These types of emerging approaches that clarify the meaning of mapped selection functions and allow for translating models to quantities such as the utilization distribution hold substantial promise toward advancing the utility of HSAs for conservation and management. The ability to produce estimates of population‐level utilization distributions from selection functions will allow managers and conservation practitioners to more easily assess the value of specific locations to a species, and we strongly recommend that these approaches begin to be implemented by those hoping to use HSAs to inform conservation and management. We note that, to date, the current applications of this utilization distribution approach from fitted SSFs have used estimated coefficients without any consideration of their uncertainty. Researchers could quantify uncertainty in these utilization distributions using a parametric bootstrap, whereby coefficient estimates were combined with their standard errors to repeatedly sample from a normal distribution and a separate utilization distribution was produced for each sample to quantify uncertainty. Producing these maps can be computationally expensive and so this bootstrap approach might be intractable, therefore the incorporation and presentation of uncertainty is an area that needs further exploration.

Researchers conducting HSAs vary widely in how they use results to create maps, leading to highly variable and often erroneous interpretation (Morris et al. 2016); there should be a high level of concordance between how a map is evaluated and how it is graphically displayed. Maps that are intended to depict “suitable habitat” and “unsuitable habitat” for conservation planning by categorizing continuous predictions should do so by assessing the cumulative percentage of selection that captures a defined percentage threshold (e.g., 80%, 85%, 90%, 95%) of habitat selection (Holbrook et al. 2017); this threshold should be chosen together with resource managers and decision makers in light of risks to the study species and the conservation question.

Although maps are often the desired endpoint for those conducting HSAs, researchers typically are interested in making direct inference to the effect of environmental covariates on habitat selection through the direction and magnitude of the regression coefficients. Fieberg et al. (2021) recently provided a thorough overview of how to interpret coefficients in HSAs. We briefly summarize some of their main points. For general, qualitative inference, the direction of coefficients estimated in a selection function (i.e., positive or negative) indicates, for continuous covariates, whether an environmental covariate was selected for (i.e., larger values of the covariate are more likely to show up in the used sample relative to the available sample) or avoided (smaller values of the covariate are more likely to show up in the used sample relative to the available sample). For categorical covariates, the coefficients reflect the ratio of used to available locations for a particular category relative to the ratio of used to available location for a reference level. As with any regression, the confidence in the direction of the effect can be evaluated using coefficient uncertainty (i.e., its standard error or using confidence intervals or Bayesian credible intervals). Despite the use of logistic regression, these regression coefficients cannot be interpreted as log‐odds ratios, but rather provide inference to relative intensities of use or relative selection strength (Lele et al. 2013, Avgar et al. 2017, Fieberg et al. 2021). One can simply present the coefficients themselves, or if there is interest in direct quantification of how the change in a specific covariate influences the relative strength of selection, researchers can take the ratio of predicted selection function values with different values of the covariate of interest (Avgar et al. 2017, Fieberg et al. 2021). Fieberg et al. (2021) further show how one can make direct inference to the relative chance of finding an animal in different land‐use categories. They achieve this by taking the ratio of the sum of the estimated selection function (i.e., $w\left(x\right)=\exp(x\beta$)) for all available points falling in one category to the sum of the estimated selection function for all available points falling in another category. Although for selection functions fit without conditional availability, this approach is straightforward, a more complex procedure is required for similar inference from SSFs because of the conditional nature of availability (e.g., Signer et al. 2017). Regardless, this approach is powerful for translating selection functions into quantities that are directly relevant for conservation and management (i.e., the relative amount of time an animal is estimated to spend in a habitat unit with some specific value of covariates). Furthermore, the approach outlined by Fieberg et al. (2021) should clarify the often apparently contradictory finding that a land cover category where the animal spent the majority of their time may have a negative coefficient. Their approach would show a greater chance of finding the animal in the more frequently used land cover type. Standardizing covariates by subtracting the mean value and dividing by the standard deviation can facilitate direct comparison of coefficient magnitudes and also facilitate interpretation from relative selection strength estimates (Schielzeth 2010). Such standardization also often helps with convergence when fitting statistical models. Further recommendations on interpreting selection coefficients and visualizing the change in relative selection strength across different habitat values are provided by Avgar et al. (2017) and Fieberg et al. (2021), and we direct readers to these sources for a thorough treatment of this topic.

### Model selection, evaluation and validation

Model selection and assessment are fundamental to ecological studies relying on statistical inference. Researchers use model selection to evaluate the relative strength of a set of models, representing alternative hypotheses. Model assessment explores whether a model can adequately reproduce observed data, therefore characterizing the model's predictive reliability. It is common for researchers applying HSAs to fit and compare multiple models, as well as seek to evaluate the adequacy of their models. As with any ecological study, model selection is routinely based on balancing a bias‐variance trade‐off (larger models tend to have less bias but higher variance), based on the data and model set (Burnham and Anderson 2002). Both fixed‐effects and mixed‐effects models, fitted in a likelihood framework, are commonly compared using information‐theoretic criteria (e.g., AIC, Bayesian Information Criteria [BIC]; Boyce et al. 2002, Hebblewhite and Merrill 2008); AIC aims to optimize asymptotic efficiency (expected predictive accuracy) and BIC to optimize consistency in identifying a correct model (Aho et al. 2014). HSAs conducted in a Bayesian framework have been compared by the Deviance Information Criterion (Thomas et al. 2006), however there is a wide number of options that compare discrete and continuous model sets (Hooten and Hobbs 2015). Model selection and ranking depend on the model set. Therefore, the model set requires justification. However, seeking model parsimony is not a necessity; large satellite‐based datasets (e.g., GPS) can make the bias‐variance trade‐off effectively irrelevant (leading to the most complicated model routinely being selected as the top model), such that ecological inference is more practically done based on parameter estimates and their uncertainty from a global model (e.g., Northrup et al. 2016). This approach is philosophically appealing in habitat‐selection studies as it places emphasis on a single complex model, including factors known and hypothesized to be important, which are not arbitrarily removed, as in common model‐selection procedures (Giudice et al. 2012, Harrell 2015). Furthermore, as HSAs often use few base products (i.e., satellite imagery) to develop a suite of correlated covariates, model‐selection procedures can turn into unsatisfying tests among covariates that largely represent the same ecological process, such as slope vs. terrain ruggedness. A thorough discussion on model building and multimodel inference is provided by Fieberg and Johnson (2015).

Once a model or set of models has been identified, the focus should be on model assessment. HSAs routinely use their fitted models to predict the relative selection strength over a study region, and often use these predictions as data in subsequent analyses (e.g., Nielsen et al. 2006, Northrup et al. 2012*b*, DeCesare et al. 2014, Ditmer et al. 2018). Whether these predictions should be considered reliable depends on the model's predictive performance. Because selection functions are often fitted with logistic regression, to approximate a point process model, there is confusion in the applied literature on how to assess model performance. Methods commonly used to evaluate logistic regression models (e.g., AUC) are not appropriate for selection functions fitted with logistic regression. Guidance on selection function evaluation is detailed in several papers (e.g., Boyce et al. 2002, Johnson et al. 2006, Wiens et al. 2008, for a review on model evaluation variability in HSAs, see Morris et al. 2016). Briefly, their suggestions are to map the selection function (i.e., $w\left(x\right)=\exp\left(x\beta \right)$), divide the landscape into bins, and compare the proportion of observed within‐sample or out‐of‐sample data to expected by simple linear regression or Spearman‐rank correlations; the ideal model would have a 1:1 linear relationship with an intercept of 0 and slope of 1. These authors suggest withholding within‐sample data, and researchers often will withhold portions of the dataset from each individual. However, Roberts et al. (2017) showed that such an approach can lead to optimistic assessments of predictive performance, and a more realistic cross‐validation measure can be obtained by withholding entire individuals. Importantly, there is no value in assessing a model's ability to predict available locations, as these are simply a computational convenience. An alternative approach to the cross‐validation procedures discussed above, is to characterize the probability of concordance between predictions and observed locations by Kendall's c statistic (Aldridge et al. 2012). Ideally, out‐of‐sample data are used, which are independent from the data used to fit the model (Coe et al. 2011). Cross‐validation procedures should consider important structuring, such as individual, spatial, and temporal dimensions (Roberts et al. 2017). Fieberg et al. (2018) suggested an approach referred to as used habitat calibration plots, which, instead of focusing on the model's ability to differentiate between used and available locations, assessed how well they described the characteristics of used locations. This approach aids in identifying missing covariates and can help to assess a model's transferability to new areas. Bayesian models can be evaluated by a range of goodness‐of‐fit procedures (Conn et al. 2018), such as using a posterior predictive check (see Northrup et al. 2015 for an example in HSAs); similar approaches can also be applied using frequentist approaches (Waller et al. 2003).

Predicting beyond the sampled study region should be done with extreme caution, and results should be treated with skepticism. First, habitat availability must be assumed to be equivalent between the study area and the new region, which is highly unlikely (Paton and Matthiopoulos 2016). Second, unless many individuals are sampled, and individual‐level variation is explicitly considered in model fitting and evaluation, predictions are likely to underestimate the variability of habitat selection to unsampled individuals. Alternately, predictions out of the study system could form the basis for hypothesis tests (e.g., Houlahan et al. 2017). For HSAs to provide robust inference on ecological and evolutionary processes, they need to be able to predict these processes in out‐of‐sample and out‐of‐system data. But this is clearly not an invitation to transfer existing models out‐of‐system; rather an invitation to challenge and validate models not with in‐sample or in‐system hold‐out‐data, but with out‐of‐sample and out‐of‐system data to better assess generalizable knowledge about selection processes.

Many HSAs are aimed at developing a predictive model to provide management guidance for a population. Current model building strategies that are prevalent in the literature may commonly lead to poor predictive models. Future studies with the goal of prediction should consider a synthetic approach to model fitting and assessment based on statistical regularization techniques (Hastie et al. 2001). Briefly, many of these techniques are able to balance the bias‐variance trade‐off continuously, as opposed to discrete model‐selection procedures in which variables are included or not. While large complex models with many non‐zero effects are likely to minimize bias, they are also likely to have high variance. Conversely, simple models with few variables may have high bias and low variance. Techniques that shrink parameters toward zero (a form of model parsimony or simplification) can often be used to improve predictive performance compared with discrete model‐selection approaches (Gerber and Northrup 2020). The amount of shrinkage can be determined by cross‐validation procedures.

## Conclusions and Future Directions

Selection functions have become the standard for assessing the process of habitat selection by animals. This approach has been applied most often to larger mammals that can carry telemetry devices, but with emerging technologies such as hydroacoustic arrays, MOTUS networks for smaller birds, and satellites such as Icarus (https://www.icarus.mpg.de/en), we will soon have the capacity to track nearly any vertebrate species. The last two decades have seen an array of conceptual and methodological advances in HSAs. As our review shows, HSAs provide an incredible breadth of opportunities for understanding ecological and evolutionary processes, equaled by the number of challenges they present. It is not reasonable to expect any single study to address all the issues and opportunities discussed here, but it is critical that researchers acknowledge the complex processes underlying data used in HSAs and endeavor to create a reproducible model. By addressing these complexities or attempting to design studies such that they are adequately controlled for, inference from HSAs will be more robust and reliable.

With continual development of new statistical models and improvements in remote sensing, animal‐borne sensors and computational power, we foresee numerous opportunities and advances just on the horizon and discuss a few of these here. First, combining GPS radio collars with animal‐borne sensors (Lynch et al. 2013, Pagano et al. 2018) will begin to provide unprecedented insight into how habitat selection relates to more direct measures of animal behavior, and how these processes ultimately influence individual foraging and reproductive success. Similarly, incorporating movement directly into HSAs will continue to improve inference, but also will bring new challenges as we become closer to sampling the continuous paths of animals. Because most studies of habitat selection require the handling of animals, therefore providing the opportunity for obtaining genetic samples, combining HSAs with genetic and genomic methods to elucidate the evolutionary underpinnings of habitat‐selection patterns will become more common (Shafer et al. 2016) but bring with it new inferential and data management challenges. Decreasing cost of collars will begin to make population‐wide and multispecies tagging studies a reality, which will open the door for more robust analyses looking at interspecific and intraspecific interactions. These types of data will also require continual methodological and computational advances to deal with increasingly large datasets. Over the past 20 yr, HSAs have emerged as the dominant analytical framework to investigate the behavior that generates the pattern of animal space use. As techniques become refined and particular analytical approaches themselves are selected for or against, the underlying theory upon which HSAs are based remains steadfast. Our aim here was to cast back across these last 20 yr and summarize the state of HSAs today; with this review we hope to create a more coherent conversation going forward over the next 20 yr to enable those conducting HSAs to acknowledge structural assumptions, develop clear *a priori* hypotheses, and subsequently execute technically robust analyses. This will enhance the potential for such analyses to be integrated into future syntheses and meta‐analyses such that the inference we can derive about ecological and evolutionary processes is robust, repeatable, and generalizable.
